# Identifying the effect of vancomycin on health care–associated methicillin-resistant *Staphylococcus aureus* strains using bacteriological and physiological media

**DOI:** 10.1093/gigascience/giaa156

**Published:** 2021-01-09

**Authors:** Akanksha Rajput, Saugat Poudel, Hannah Tsunemoto, Michael Meehan, Richard Szubin, Connor A Olson, Yara Seif, Anne Lamsa, Nicholas Dillon, Alison Vrbanac, Joseph Sugie, Samira Dahesh, Jonathan M Monk, Pieter C Dorrestein, Rob Knight, Joe Pogliano, Victor Nizet, Adam M Feist, Bernhard O Palsson

**Affiliations:** Department of Bioengineering, University of California, 9500 Gilman Dr, La Jolla, CA 92093, USA; Department of Bioengineering, University of California, 9500 Gilman Dr, La Jolla, CA 92093, USA; Division of Biological Sciences, University of California, San Diego, 9500 Gilman Dr, La Jolla, CA 92093, USA; Collaborative Mass Spectrometry Innovation Center, University of California San Diego, 9500 Gilman Dr, La Jolla, CA 92093, USA; Skaggs School of Pharmacy and Pharmaceutical Sciences, University of California San Diego, 9500 Gilman Dr, La Jolla, CA 92093, USA; Department of Bioengineering, University of California, 9500 Gilman Dr, La Jolla, CA 92093, USA; Department of Bioengineering, University of California, 9500 Gilman Dr, La Jolla, CA 92093, USA; Department of Bioengineering, University of California, 9500 Gilman Dr, La Jolla, CA 92093, USA; Division of Biological Sciences, University of California, San Diego, 9500 Gilman Dr, La Jolla, CA 92093, USA; Department of Pediatrics, University of California San Diego, 9500 Gilman Dr, La Jolla, CA 92023, USA; Collaborative to Halt Antibiotic-Resistant Microbes (CHARM), Department of Pediatrics, University of California San Diego, 9500 Gilman Dr, La Jolla, CA 92093, USA; Department of Pediatrics, University of California San Diego, 9500 Gilman Dr, La Jolla, CA 92023, USA; Collaborative to Halt Antibiotic-Resistant Microbes (CHARM), Department of Pediatrics, University of California San Diego, 9500 Gilman Dr, La Jolla, CA 92093, USA; Division of Biological Sciences, University of California, San Diego, 9500 Gilman Dr, La Jolla, CA 92093, USA; Department of Pediatrics, University of California San Diego, 9500 Gilman Dr, La Jolla, CA 92023, USA; Collaborative to Halt Antibiotic-Resistant Microbes (CHARM), Department of Pediatrics, University of California San Diego, 9500 Gilman Dr, La Jolla, CA 92093, USA; Department of Bioengineering, University of California, 9500 Gilman Dr, La Jolla, CA 92093, USA; Collaborative Mass Spectrometry Innovation Center, University of California San Diego, 9500 Gilman Dr, La Jolla, CA 92093, USA; Skaggs School of Pharmacy and Pharmaceutical Sciences, University of California San Diego, 9500 Gilman Dr, La Jolla, CA 92093, USA; Center for Marine Biotechnology and Biomedicine, Scripps Institution of Oceanography, University of California San Diego, 9500 Gilman Dr, La Jolla, CA 92093, USA; Center for Microbiome Innovation, University of California San Diego, 9500 Gilman Dr, La Jolla, CA 92093, USA; Department of Bioengineering, University of California, 9500 Gilman Dr, La Jolla, CA 92093, USA; Department of Pediatrics, University of California San Diego, 9500 Gilman Dr, La Jolla, CA 92023, USA; Center for Microbiome Innovation, University of California San Diego, 9500 Gilman Dr, La Jolla, CA 92093, USA; Department of Computer Science and Engineering, University of California San Diego, 9500 Gilman Dr, La Jolla, CA 92093, USA; Division of Biological Sciences, University of California, San Diego, 9500 Gilman Dr, La Jolla, CA 92093, USA; Skaggs School of Pharmacy and Pharmaceutical Sciences, University of California San Diego, 9500 Gilman Dr, La Jolla, CA 92093, USA; Department of Pediatrics, University of California San Diego, 9500 Gilman Dr, La Jolla, CA 92023, USA; Collaborative to Halt Antibiotic-Resistant Microbes (CHARM), Department of Pediatrics, University of California San Diego, 9500 Gilman Dr, La Jolla, CA 92093, USA; Center for Microbiome Innovation, University of California San Diego, 9500 Gilman Dr, La Jolla, CA 92093, USA; Department of Bioengineering, University of California, 9500 Gilman Dr, La Jolla, CA 92093, USA; Novo Nordisk Foundation Center for Biosustainability, Technical University of Denmark, Kemitorvet, Building 220, 2800 Kongens, Lyngby, Denmark; Department of Bioengineering, University of California, 9500 Gilman Dr, La Jolla, CA 92093, USA; Department of Pediatrics, University of California San Diego, 9500 Gilman Dr, La Jolla, CA 92023, USA; Center for Microbiome Innovation, University of California San Diego, 9500 Gilman Dr, La Jolla, CA 92093, USA; Novo Nordisk Foundation Center for Biosustainability, Technical University of Denmark, Kemitorvet, Building 220, 2800 Kongens, Lyngby, Denmark

## Abstract

**Background:**

The evolving antibiotic-resistant behavior of health care–associated methicillin-resistant *Staphylococcus aureus* (HA-MRSA) USA100 strains are of major concern. They are resistant to a broad class of antibiotics such as macrolides, aminoglycosides, fluoroquinolones, and many more.

**Findings:**

The selection of appropriate antibiotic susceptibility examination media is very important. Thus, we use bacteriological (cation-adjusted Mueller-Hinton broth) as well as physiological (R10LB) media to determine the effect of vancomycin on USA100 strains. The study includes the profiling behavior of HA-MRSA USA100 D592 and D712 strains in the presence of vancomycin through various high-throughput assays. The US100 D592 and D712 strains were characterized at sub-inhibitory concentrations through growth curves, RNA sequencing, bacterial cytological profiling, and exo-metabolomics high throughput experiments.

**Conclusions:**

The study reveals the vancomycin resistance behavior of HA-MRSA USA100 strains in dual media conditions using wide-ranging experiments.

## Background

The prevalence of methicillin-resistant *Staphylococcus aureus* (MRSA) infections such as bacteremia differs around the world, and it is one of the leading causes of nosocomial infections worldwide [1]. Health care–associated MRSA (HA-MRSA) is a subset of MRSA strains that often circulate in health care settings such as hospitals and dialysis centers [2–4]. The USA100 strain is a HA-MRSA that shows high resistance to a wide range of antibiotics such as macrolides, fluoroquinolones, and lincosamides [5, 6]. Moreover, these strains are considered to display vancomycin-resistant and intermediate phenotypes [7]. Over the past 4 decades, vancomycin has been the antibiotic of choice to treat MRSA. However, by the 1990s vancomycin-intermediate strains (VISA) had already begun to emerge [8]. In 2002, the United States reported the first case of vancomycin-resistant *S. aureus* [9]. To understand the genetic and phenotypic basis for the emergence of this resistance, we collected multi-omic data on HA-MRSA strains D592 (daptomycin-susceptible) and its descendent D712 (daptomycin-nonsusceptible) previously collected from a patient with prolonged and persistent MRSA bacteremia for 21 days [10, 11]. Vancomycin is one of the few drugs that works against daptomycin-susceptible and daptomycin-nonsusceptible strains.

Cation-adjusted Mueller-Hinton broth (CAMHB) is a standard medium for quantitative procedures for susceptibility testing in microbiology laboratories worldwide [12]. CAMHB is commonly used for antibiotic susceptibility testing because it is enriched with the divalent ions Ca^2+^ and Mg^2+^. The presence of divalent ions affects the stability of antibiotics or mode of action of antibiotics, which in turn greatly affects the minimal inhibitory concentration (MIC) values. Roswell Park Memorial Institute (RPMI) 1640 media is among the best media with which to mimic human physiology [13–15].

The present study is focused on exploring the effect of vancomycin on HA-MRSA USA100 D712 and D592 strains in bacteriological (CAMHB) and physiological (RPMI+10%LB) media. The MIC value of D592 decreased from 2 µg/mL R10LB to 1 µg/mL in CAMHB in the presence of vancomycin. Furthermore, for the D712 strain, the MIC value decreased from 2 µg/mL in R10LB to 0.96 µg/mL in CAMHB. Although these MIC values fall below the clinically defined vancomycin resistance levels (MIC ≥ 16 µg/mL), vancomycin treatment was not able to clear the bacteremia caused by these isolates [10]. Here we interrogated the response of HA-MRSA strains to the subinhibitory concentration of vancomycin using growth curves, RNA sequencing (RNA-seq), bacterial cytological profiling (BCP), and exo-metabolomics (high-performance liquid chromatography [HPLC] and liquid chromatography–mass spectroscopy [LC/MS]). Together, our data provide an in-depth look into vancomycin response by simultaneously tracking gene expression (RNA-seq), cell morphology (BCP), and changes in the chemical composition of the media (HPLC and LC/MS).

## Methods

The methods used in the present study are validated in our previous articles [14, 16].

### Culture and growth conditions

**Figure 1:**
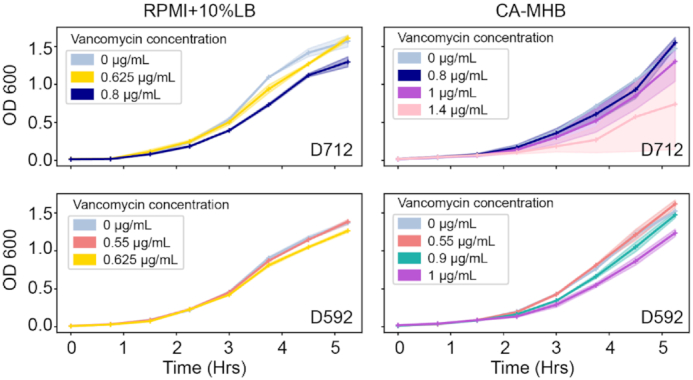
Growth curve for *Staphylococcus aureus* D592 and D712 strains in presence of vancomycin at various subinhibitory concentrations in CA-MHB and R10LB media. The shaded areas in the graph represents the confidence interval.

We used standard bacteriological and physiological media to identify the effect of vancomycin on USA100 strains. The standard bacteriological media includes Mueller-Hinton broth (Sigma-Aldrich, St Louis, MO, USA) and is supplemented with 25 mg/L Ca ^2+^ and 12.5 mg/L Mg^2+^ (CAMHB). The physiological media includes RPMI 1640 as eukaryotic cell culture medium (Thermo Fisher Scientific, Waltham, MA, USA), which was supplemented by 10% LB (R10LB). The broth microdilution assay was performed to check the MIC of vancomycin in both the media conditions. Both the USA100 HA-MRSA, i.e., D592 and D712, were grown overnight. Furthermore, overnight-grown samples were diluted starting from OD600 of 0.01 through fresh media to OD600 of 0.4 at 37°C. The preculture was again diluted to OD600 of 0.01 by fresh media in the absence of vancomycin. Overall growth was monitored and OD600 readings were taken for 5.25 hr at every 45 min. For all subsequent experiments, and based on preliminary growth analysis, D592 was exposed to 0, 0.55, 0.9, and 1.0 µg/mL of vancomycin in CAMHB medium and 0, 0.55, and 0.625 µg/mL in RPMI. D712 was exposed 0, 0.8, 1, and 1.4 µg/mL in CAMHB and 0, 0.625, and 0.8 µg/mL in RPMI. The growth curve is provided in Fig.   1. Concentrations of vancomycin shown in Fig. 1 were chosen from a larger range of antibiotic concentrations based on the pattern of growth inhibition. To get reasonable and reproducible profiles in all downstream analyses, final assay concentrations were based on the constraint of ≤50% growth inhibition relative to the untreated control of the strain of interest in the media of interest rather than multiples of the respective MIC. Preliminary growth curves were performed in at least biological duplicate, depending on the difficulty in finding reasonable growth inhibition, and final assay experiments were performed in biological duplicate. We were also interested in looking at overlapping concentrations for each strain, which is why there is an additional antibiotic concentration in the CA-MHB conditions but not in the RPMI+10%LB conditions.

### Complementary DNA library preparation and RNA sequencing

For RNA-seq, the tubes containing 3 mL samples were taken after 3 hr and added to a tube containing 6 mL RNAprotect and centrifuged after incubation. The 3-hr time point for RNA-seq was chosen to allow for ∼4 doublings of the bacterial population in the presence of vancomycin, providing time for the antibiotic to have a robust effect on the transcriptional and phenotypic response of the bacteria. All the experiments were performed in 2 iological replicates. The “Quick RNA Fungal/Bacterial Microprep” kit (Zymo Research, Irvine, USA) was used for the RNA extraction from the pellet cells. During the RNA purification, the mechanical lysis was performed with the Roche MagNa Lyser instrument, while DNA was removed through DNase I treatment. The Illumina Ribo-Zero kit was used to remove the ribosomal RNA. The quality of RNA was checked with the Agilent Bioanalyzer instrument. Furthermore, the complementary DNA library was constructed before sequencing through a KAPA Stranded RNA-seq Library Preparation Kit. Last, the RNA fragmentation, sequencing adapter ligation, and library amplification were performed. The generated complementary DNA libraries were sent for Illumina sequencing on the HiSeq 4000 platform.

### RNA sequencing analysis

**Figure 2:**
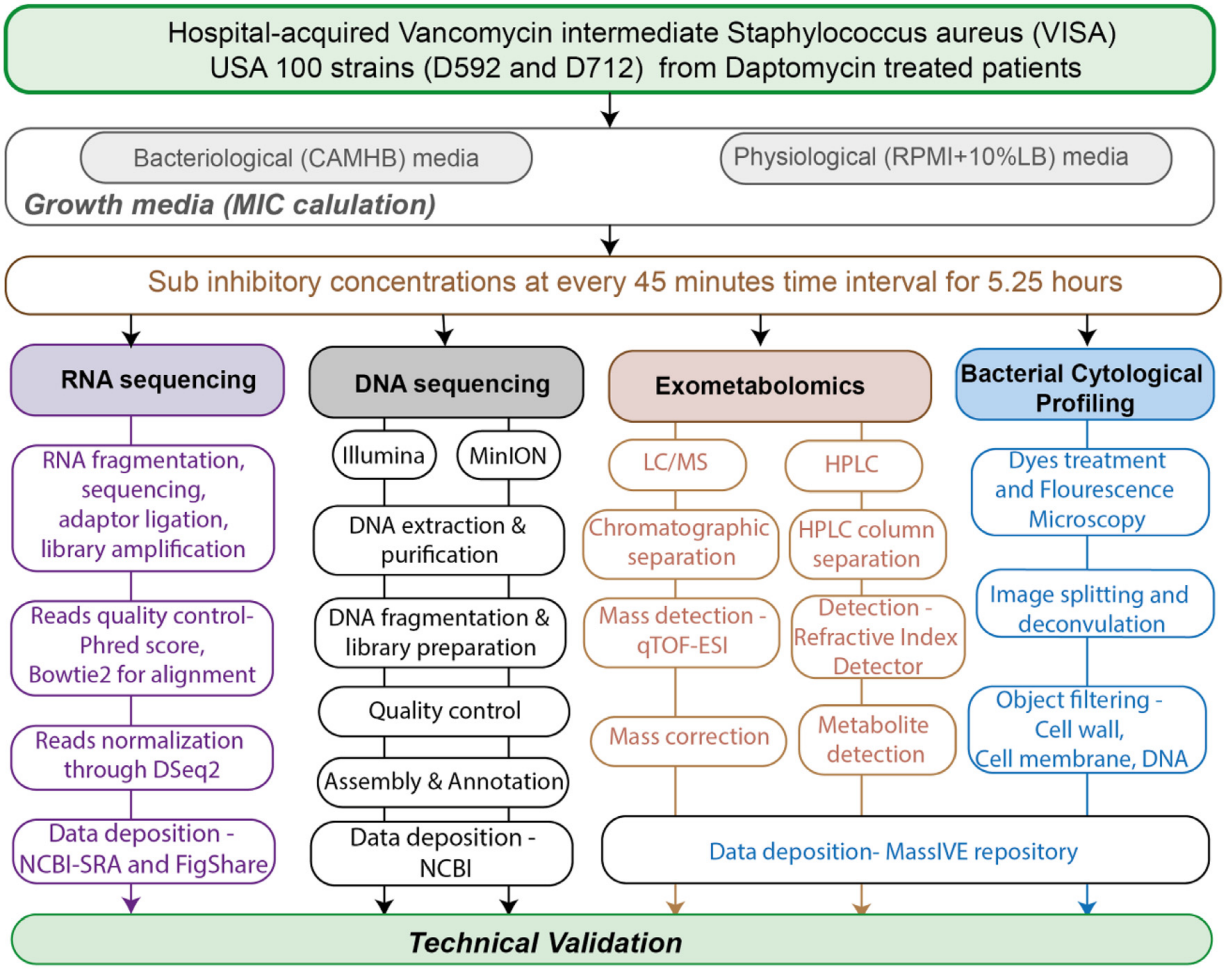
Diagram depicting the methodology of high-throughput approaches used to profile the *Staphylococcus aureus* D592 and D712 in presence of vancomycin. qTOF-ESI: quadrupole time-of-flight electrospray ionization.

For Illumina sequencing, the Phred quality scores were generated using the Fastqc package [17]. The alignment of the raw reads was done for D712 and D592 genomes using Bowtie2 [18, 19], and FastQC [20] to calculate alignment percentage. Furthermore, the DESeq2 package was used to normalize the aligned reads to transcripts per million (TPM). Last, the technical validation was done through principal component analysis (PCA) using the sklearn package [21, 22, 23]. The summary steps are provided in Fig. 2.

### DNA sequencing and genome assembly

The reference genome of D592 and D712 was sequenced using an Illumina Hiseq 4000 (paired-end, 100/100 bp reads) and Nanopore MinION to 50× and 60× coverage. First, for the Illumina sequencing, the genomic DNA was prepared with the Zymo Research Quick-DNA Fungal/Bacterial Microprep Kit. However, the Kapa Biosystems HyprePlus kit was used to construct the libraries. Second, for the MinION sequencing, the genomic DNA was prepared through the CTAB method. Furthermore, the Oxford Nanopore Rapid Barcoding Kit was used to construct the libraries. The quality control steps involved the removal of unincorporated primers, PCR primers, and adapters. The assembly step involved Unicycler 0.4.2 in the “default” mode for assembling 02 contigs (genome and plasmid). Last, the annotation was performed through the NCBI Prokaryotic Genome Annotation Pipeline (PGAP) v4.11.

### Bacterial cytological profiling

After 3 hr of treatment, samples were removed for fluorescence microscopy, as previously described with slight modifications [24–26]. The 3-hr time point for BCP was chosen to allow for ∼4 doublings of the bacterial population in the presence of vancomycin, providing time for the antibiotic to have a robust effect on the transcriptional and phenotypic response of the bacteria. All the experiments were performed in 2 biological replicates. In brief, 8 µL treated samples were added to tubes containing 2 µL dye mix (10 µg/mL DAPI, 2.5 µM SYTOX Green, 60 µL/mL FM4–64 in 1× T-base). The samples were then spotted onto an agarose pad slide (20% media, 1.2% agarose) for microscopy. Imaging was performed on an Applied Precision DV Elite epifluorescence microscope with a CMOS camera, with excitation and exposure times kept constant for all images (TRITC/Cy-5 = 0.025 s,  FITC/FITC = 0.01 s,  DAPI/DAPI = 0.015 s).

FIJI (ImageJ 1.51w) and Adobe Photoshop (2015.1) were used to adjust deconvolved images to decrease background in FM4–64 and DAPI channels to ensure proper identification of cell and DNA objects. Raw and deconvolved images were then further processed using a custom CellProfiler 3.0 pipeline that individually thresholded and filtered DAPI and FM4–64 channels to obtain segmentation masks for key cellular features such as the cell membrane, DNA, and entire cell, for a total of 5,285 features [27, 28]. Feature selection was applied prior to analysis to create a subset of relevant features and minimize redundancy. The summary of the processing steps is presented in Fig. 2.

### Untargeted liquid chromatography–mass spectrometry data acquisition

At the same time that samples of HA-MRSA USA100 D592 and D7128 were taken for OD600 measurements, ∼400  µL was collected from each replicate of all growth conditions and syringe-filtered using 0.22- µm disc filters (Millex-GV, MilliporeSigma) to remove the cells from the spent media. Exo-metabolomics data were taken every 45 minutes from T0 to T4.5 hr, in coordination with the bacterial doubling time. Filtered samples were immediately placed on dry ice and then stored at −80°C until LC/MS was performed. The LC/MS platform used an UltiMate3000 HPLC system (Thermo Scientific) paired to a Maxis Impact (Bruker Daltonics) quadrupole-time-of-flight mass spectrometer. Filtered media from the RPMI+10% LB cultures were injected onto the LC at a volume of 5 µL, and filter media from CAMHB cultures were injected at a volume of 2 µL. All samples were injected onto a Kinetex 2.6-µm polar-C18 reverse-phase column (Phenomenex). The column temperature was maintained at 30°C. All samples were taken in 2 biological replicates.

For chromatographic separations, mobile phase A was LC/MS-grade water modified with 0.1% formic acid and mobile phase B was LC/MS-grade acetonitrile modified with 0.1% formic acid. Samples were injected at 95% A/5% B and at 1 minute the gradient was ramped to 65%A/35%B over the next 4 minutes. The solvent composition was stepped up to 0%A/100%B and held for 1 minute before being restored to 95%A/5%B for equilibration prior to injection of the subsequent sample.

Eluent from the HPLC was sprayed into the Maxis Impact mass spectrometer via an Apollo II electrospray ionization (ESI) source. The mass spectrometer was controlled using v4.0.15 and the LC/MS sequence program was controlled using Hystar v3.2. During the sample introduction into the mass spectrometer, the ESI source was configured to have a nebulizer gas pressure of 2 bar, drying gas flow rate of 9 L/min, and a drying gas temperature of 200°C. The mass spectrometer's inlet capillary voltage was set at 3,500 V with an endplate offset of 500 V. The mass spectrometer ion transfer optics were set to the following: ion funnel 1,250 Vpp (volts peak-to-peak), ion funnel 2,250 Vpp, transfer hexapole RF 100  Vpp, quadrupole ion energy 5  eV, and collision quadrupole energy of 5  eV. The collision quadrupole RF was stepped across 4 voltages per scan: 450, 550, 800, and 1100  Vpp. The collision cell transfer time was stepped across 4 values per scan: 70, 75, 90, and 95  µsec. TOF pre-pulse storage was fixed at 7.0 µsec. The mass spectrometer scan rate was fixed at 3 Hz.

Prior to analysis, the mass spectrometer was externally calibrated using a sodium formate solution that was prepared by adding 100  μL of 1  M NaOH and 0.2% formic acid into 9.9 mL of a 50%/50% water and isopropanol mixture. During mass spectrometric data acquisition, hexakis (1H,1H,2H-difluoroethoxy)-phosphazene (SynQuest Labs, Inc.) was used as a “lock mass” internal calibrant (positive mode: *m*/*z* 622.028960; C_12_H_19_F_12_N_3_O_6_P_3_+; negative polarity: ion *m*/*z* 556.001951; C_10_H_15_F_10_N_3_O_6_P_3_−). Subsequent to data acquisition, the lock mass was used to apply a linear mass correction to all mass spectra using Bruker Daltonics Compass Data Analysis software (ver. 4.3.110). Lock mass–corrected data files were converted from the proprietary format (.d) to the mzXML open data format. All data herein were deposited to MassIVE (http://massive.ucsd.edu). The brief methodology is provided in Fig. 2.

### Targeted high-performance liquid chromatography

**Figure 3:**
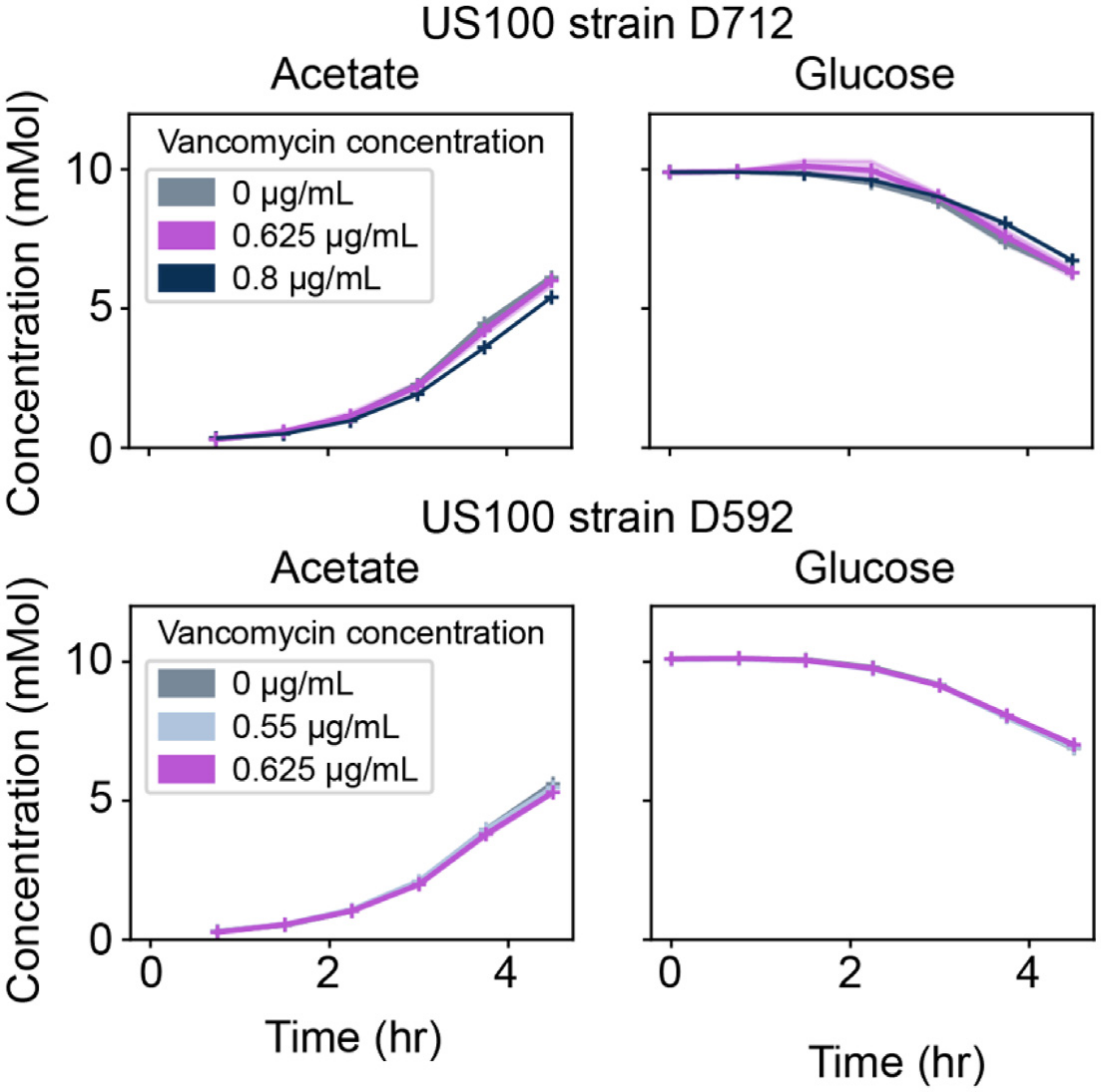
HPLC-derived quantitative time-course exo-metabolomics measurements for *Staphylococcus aureus*D592 and D712 cells exposed to various antibiotic concentrations in RPMI + 10%LB and CAMHB. Here, we show the absolute calibrated concentrations of acetate and D-glucose in RPMI + 10%LB media type. However, the HPLC time-course exo-metabolomics measurements for *S. aureus* D592 and D712 cells in the presence of different concentrations of vancomycin in CAMHB media are not shown because the differences were too slight.

The HPLC experiment was performed for the detection of organic acids and carbohydrates. All the samples were filtered and collected every 45 minutes as discussed above. All the samples were taken in 2 biological replicates. The 1260 Infinity series (Agilent Technologies) HPLC system was used to load the samples using Aminex HPX-87H column (Bio-Rad Laboratories) and a refractive index detector. The overall system was run through ChemStation software. The HPLC-grade water buffered with 5  mM sulfuric acid (H_2_SO_4_) was used as a single mobile phase. At the temperature of 45°C, the 10-µL samples were injected with a flow rate of 0.5  mL/minute. The compounds such as ethanol, acetate, lactate, glucose, succinate, and pyruvate were determined by comparing the retention time with their standard graph. Finally, the resulting chromatograms and peak area integration were generated using ChemStation. The chromatograms and peaks were compared with the standard graph to detect the concentration of each compound within the samples. These final concentration values were deposited into the MassIVE database. The procedure of HPLC is depicted in Fig. **2**, while the measured concentration of 2 carbon sources in RPMI + 10%LB is provided in Fig. 3. However, the HPLC time-course exo-metabolomics measurements for *S. aureus* D592 and D712 cells in the presence of different concentrations of vancomycin in CAMHB media are not shown because the differences were too slight.

## Results and Validation

### Exclusion criteria

The data of 1.4 µg/mL subinhibitory concentration for CAMHB on the D712 strain have been excluded from all studies because the reproducibility between the samples was too low. All the data are available in public repositories.

### DNA sequencing

The reference genome *S. aureus* D712 (VFJD01000001.1) [29] and D592 (NZ_CP035791.1) [30] were submitted to NCBI. The genome coverage of reference genome *S. aureus* D712 is 60×, with a final genome size of 2,825,989 bp, while for *S. aureus* D592, the genome coverage and size are 50× and 2,820,177 bp, respectively. D712 is an evolved strain of D592. Both D592 and D712 strains were collected from the same patient before and after daptomycin treatment, respectively.

### RNA sequencing

**Figure 4:**
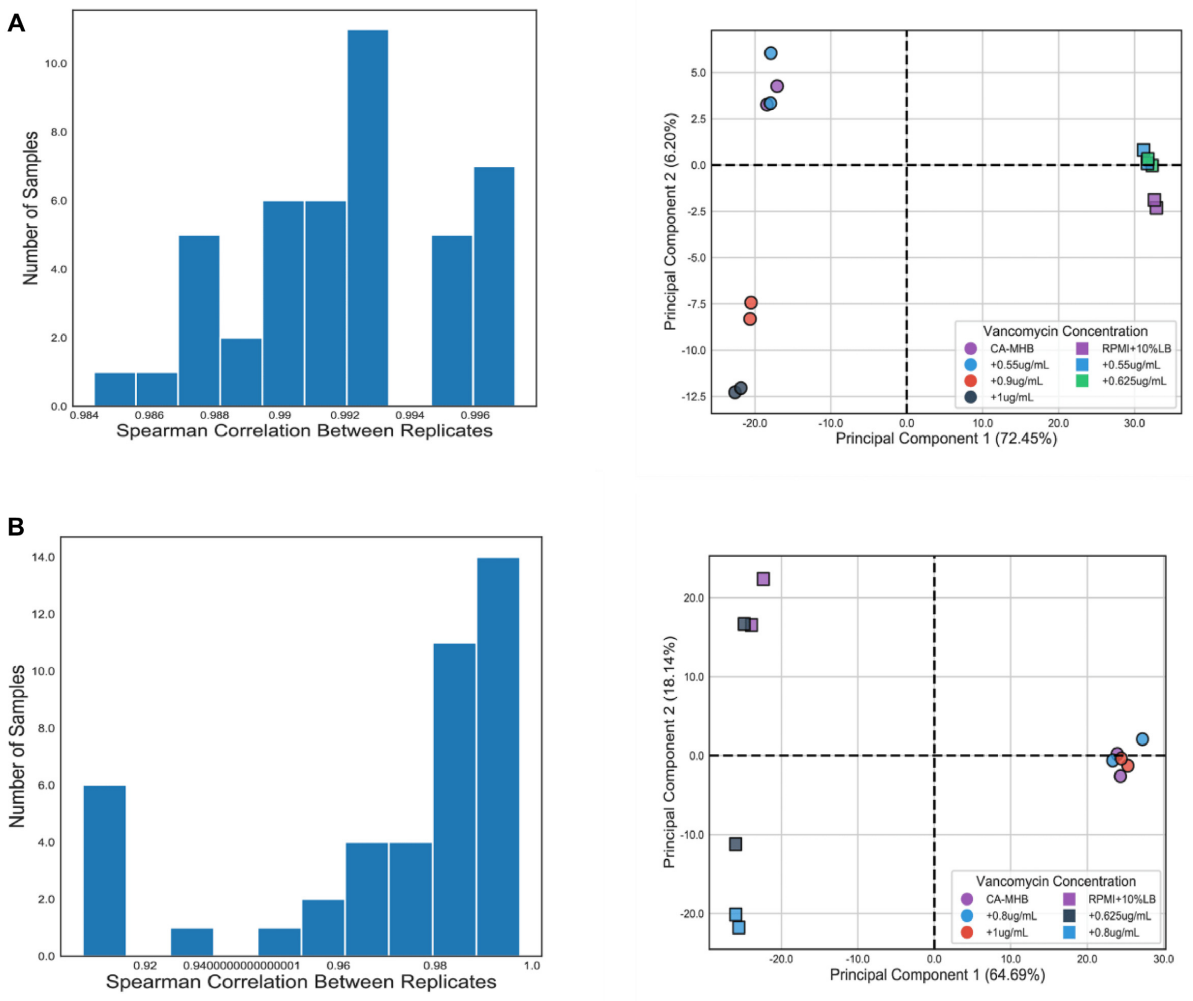
RNA-seq results. (A) Clustering of reads TPM as per Spearman correlation coefficient and PCA plot for D592 strains. (B) Clustering of reads TPM as per Spearman correlation coefficient and PCA plot for D712 strains.

First, the quality control steps were performed to remove unincorporated primers, adaptors, and detectable PCR primers. Furthermore, the sequencing reads show a mean (average) Phred score in D592 and D712 of >38.1 and >39.1, respectively. The raw fastq files were uploaded to the NCBI BioProject web platform. The alignment of reads with the reference genome in D592 and D712 gives an alignment score of 98.55% and 99.52% correspondingly. The RNA-seq results are shown in Fig. 4.

### Bacterial cytological profiling

**Figure 5:**
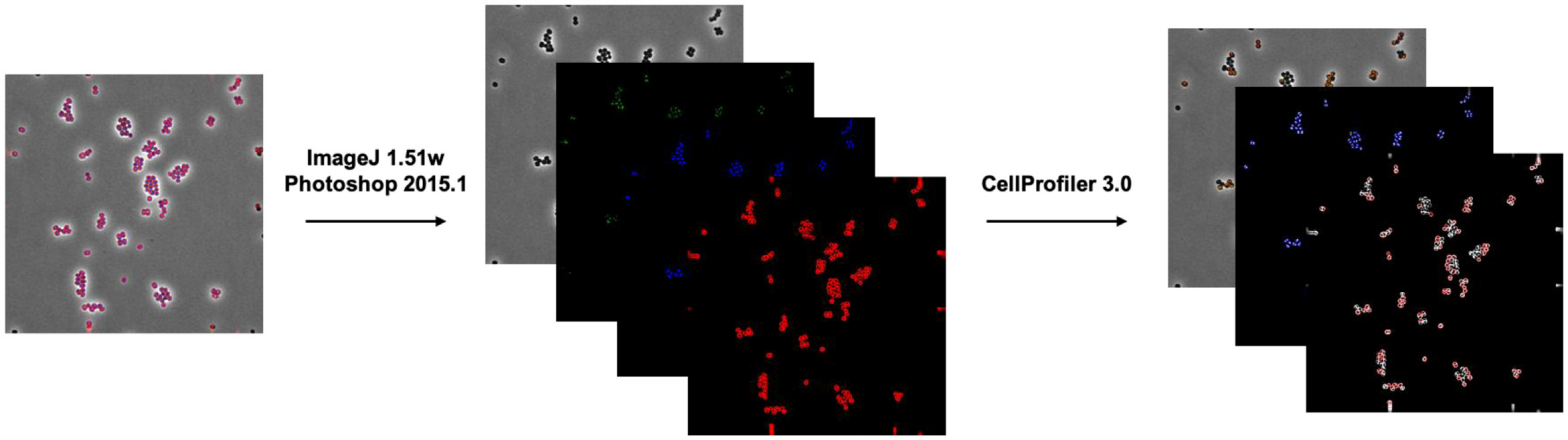
Depiction of image analysis pipeline for bacterial cytological profiling of*Staphylococcus aureus* D592 and D712 in presence of vancomycin.

Manual screening was performed on the image segmentation process of the CellProfiler. We scanned representative images through manual curation of accurate cells, object traces, and measurements. Furthermore, the cell outlines were overlaid on the corresponding related structures, e.g., DNA, cell membrane, and cell wall. Finally, the resultant files for all the cellular features were uploaded to the MassIVE repository. A representation of the image analysis pipeline for the BCP data is shown in Fig. 5.

### Untargeted liquid chromatography mass spectrometry data acquisition

For each sample, the reproducibility of global retention time and ion intensity was calculated by comparing the base peak chromatograms (BPCs) and multiple extracted ion chromatograms (EICs). The BPCs of each experimental replicate were obtained by comparing the peak intensity and reproducibility of retention time, while the EICs of the molecules were evaluated using the peak area and retention time drift of <0.1 minutes and <15% correspondingly.

### Reuse potential

Systems analysis and machine learning methods are increasingly being used to understand antibiotic resistance. The models generated by these methods, however, require a large volume of high-quality and well-curated data to be parameterized properly. The data and the associated metadata presented herein will be valuable in parameterizing many different types of models that can be used to query the underlying causes of antibiotic resistance.

The increasingly ubiquitous RNA-seq data have been used to predict the transcriptional regulatory networks in *S. aureus*, where antibodies for most regulators are not readily available [36]. These data have also been used to predict fitness and sensitivities to different antibiotics in different pathogens [37]. In parallel to RNA-seq–based approaches, metabolic modeling has also come to the forefront of the effort to understand resistance mechanisms. Combining metabolic models with machine learning methods has revealed metabolic pathways crucial for antibiotic resistance [38, 39]. These metabolic models can be further parameterized to condition-specific states with the presented exo-metabolomics data [40–42].

Last, the BCP data have been independently used to predict the various cellular subsystems that are affected by any given (known or unknown) antibiotic [25]. Together, these datasets will be used to generate models that inform us about transcriptional regulation, metabolic shifts, and morphological changes in response to antibiotic resistance.

## Data Availability

The growth-rate data are available on Figshare [31], while BCP, HPLC, and mass spectrometry data have been deposited in the MassIVE repository (MSV000085358) [32]. The complete RNA-seq pipeline can be found at Figshare [33], and Fastq files of each run have been deposited in the NCBI database (BioProject PRJNA638628) [34]. The overall summarized statistics of RNA-seq is available on Figshare [35].

## Code Availability

The complete RNA-seq pipeline used in the analysis of RNA-seq data is available on Figshare under MIT license [33].

## Abbreviations

BCP: bacterial cytological profiling; bp: base pairs; CAMHB: cation-adjusted Mueller-Hinton broth; CMOS: complementary metal–oxide–semiconductor; CTAB: cetyl trimethylammonium bromide; DAPI: 4′,6-diamidino-2-phenylindole; EIC: extracted ion chromatogram; ESI: electrospray ionization; LC/MS: liquid chromatography–mass spectroscopy; MIC: minimal inhibitory concentration; MRSA: methicillin-resistant *Staphylococcus aureus*; HA-MRSA: health care–associated methicillin-resistant *Staphylococcus aureus*; HPLC: high-performance liquid chromatography; NCBI: National Center for Biotechnology Information; NIAID: National Institute of Allergy and Infectious Diseases; NIH: National Institutes of Health; PCA: principal component analysis; RNA-seq: RNA sequencing; RPMI: Roswell Park Memorial Institute; SRA: Sequence Read Archive; TPM: transcripts per million.

## Competing Interests

The authors declare that they have no competing interests.

## Funding

This research was supported by NIH NIAID grant (1-U01-AI124316).

## Authors' Contributions

A.R. compiled and analyzed results and wrote Background, Results, Validation, Methods, and Reuse potential. S.P. analyzed RNA sequencing data and wrote Methods. H.T. performed growth experiments, analyzed BCP data, and wrote Methods. M.M. analyzed HPLC data and wrote Data Descriptor, Methods. R.S. prepared samples for RNA sequencing and wrote Methods. C.A.O. prepared samples for HPLC and wrote Methods. Y.S. analyzed HPLC data. A.L. performed growth experiments, analyzed BCP data, and wrote Methods. N.D. performed preliminary growth and MIC experiments. A.V. performed growth experiments. J.S. wrote Methods. S.D. performed growth experiments. JMM did DNA sequencing and assembly analysis. PCD, RK, JP, VN, AMF, BOP did overall supervision.

## Supplementary Material

giaa156_GIGA-D-20-00307_Original_Submission

giaa156_GIGA-D-20-00307_Revision_1

giaa156_GIGA-D-20-00307_Revision_2

giaa156_Response_to_Reviewer_Comments_Original_Submission

giaa156_Response_to_Reviewer_Comments_Revision_1

giaa156_Reviewer_1_Report_Original_Submission Jhih-Hang Jiang -- 11/6/2020 Reviewed

giaa156_Reviewer_2_Report_Original_Submission David R. Cameron -- 11/16/2020 Reviewed

giaa156_Reviewer_2_Report_Revision_1 David R. Cameron -- 11/26/2020 Reviewed
