# Supplementary material for: Identifying the effect of vancomycin on health care–associated methicillin-resistant Staphylococcus aureus strains using bacteriological and physiological media
Source: Gigascience. 2021 Jan 9;10(1):giaa156. doi: 10.1093/gigascience/giaa156 (PMC7794652; doi:10.1093/gigascience/giaa156)
Supplement: giaa156_GIGA-D-20-00307_Original_Submission [file giaa156_giga-d-20-00307_original_submission.pdf]

GigaScience

Identifying the effect of vancomycin on HA-MRSA strains using bacteriological and physiological media

--Manuscript Draft--

**Manuscript Number:**	GIGA-D-20-00307
**Full Title:**	Identifying the effect of vancomycin on HA-MRSA strains using bacteriological and physiological media
**Article Type:**	Data Note
**Funding Information:**	National Institute of Allergy and Infectious Diseases (1-U01-AI124316) — Dr Bernhard Palsson
**Abstract:**	Evolving antibiotic resistant behavior of Healthcare-associated methicillin-resistant Staphylococcus aureus (HA-MRSA) USA100 strains are of major concern. They are resistant to a broad class of antibiotics like macrolides, aminoglycosides, fluoroquinolones, and many more. The selection of appropriate antibiotic susceptibility examination media is very important. Thus, we use bacteriological (CA-MHB) as well as physiological (R10LB) media to determine the effect of vancomycin on USA100 strains. The study includes the profiling behavior of HA-MRSA USA100 D592 and D712 strains in the presence of vancomycin through various high-throughput assays. The US100 D592 and D712 strains were characterized at sub-inhibitory concentrations through growth curves, RNA sequencing, bacterial cytological profiling, and exo-metabolomics high throughput experiments. The study reveals the vancomycin resistance behavior of USA100 strains in dual media conditions using wide-ranging experiments.
**Corresponding Author:**	Bernhard Palsson<br>University of California San Diego<br>La Jolla, CA UNITED STATES
**Corresponding Author Secondary Information:**	
**Corresponding Author's Institution:**	University of California San Diego
**Corresponding Author's Secondary Institution:**	
**First Author:**	Akanksha Rajput
**First Author Secondary Information:**	
**Order of Authors:**	Akanksha Rajput<br>Saugat Poudel<br>Hannah Tsunemoto<br>Michael Meehan<br>Richard Szubin<br>Connor A. Olson<br>Yara Seif<br>Anne Lamsa<br>Nicholas Dillon<br>Alison Vrbanac<br>Joseph Sugie<br>Samira Dahesh<br>Jonathan M. Monk

Powered by Editorial Manager® and ProduXion Manager® from Aries Systems Corporation

	Pieter C. Dorrestein
	Rob Knight
	Joe Pogliano
	Victor Nizet
	Adam M. Feist
	Bernhard Palsson
**Order of Authors Secondary Information:**	
**Additional Information:**	
**Question**	**Response**
Are you submitting this manuscript to a special series or article collection?	No
**Experimental design and statistics**<br><br>Full details of the experimental design and statistical methods used should be given in the Methods section, as detailed in our Minimum Standards Reporting Checklist. Information essential to interpreting the data presented should be made available in the figure legends.<br><br>Have you included all the information requested in your manuscript?	Yes
**Resources**<br><br>A description of all resources used, including antibodies, cell lines, animals and software tools, with enough information to allow them to be uniquely identified, should be included in the Methods section. Authors are strongly encouraged to cite Research Resource Identifiers (RRIDs) for antibodies, model organisms and tools, where possible.<br><br>Have you included the information requested as detailed in our Minimum Standards Reporting Checklist?	Yes
**Availability of data and materials**<br><br>All datasets and code on which the	Yes

conclusions of the paper rely must be either included in your submission or deposited in publicly available repositories (where available and ethically appropriate), referencing such data using a unique identifier in the references and in the "Availability of Data and Materials" section of your manuscript.<br><br>Have you have met the above requirement as detailed in our Minimum Standards Reporting Checklist?	

Powered by Editorial Manager® and ProduXion Manager® from Aries Systems Corporation

Manuscript
Click here to access/download;Manuscript;VanD712_D592_R1.docx

Identifying the effect of vancomycin on HA-MRSA strains using bacteriological and physiological media

Akanksha Rajput[1], Saugat Poudel[1], Hannah Tsunemoto[2], Michael Meehan[3,4], Richard Szubin[1], Connor A. Olson[1], Yara Seif[1], Anne Lamsa[2], Nicholas Dillon[5,10], Alison Vrbanac[5,10], Joseph Sugie[2], Samira Dahesh[5,10], Jonathan M. Monk[1], Pieter C. Dorrestein[3,4,7,9], Rob Knight[1,5,8,9], Joe Pogliano[2], Victor Nizet[4,5,9,10], Adam M. Feist[1,6] & Bernhard O. Palsson[1,5,6,9†=]

Author Affiliations

1. Department of Bioengineering, University of California, San Diego, La Jolla, USA
2. Division of Biological Sciences, University of California San Diego, La Jolla, CA 92093, USA
3. Collaborative Mass Spectrometry Innovation Center, University of California, San Diego, La Jolla, California, USA
4. Skaggs School of Pharmacy and Pharmaceutical Sciences, University of California San Diego, La Jolla, CA, USA
5. Department of Pediatrics, University of California, San Diego, La Jolla, CA, USA
6. Novo Nordisk Foundation Center for Biosustainability, Technical University of Denmark, Kemitorvet, Building 220, 2800 Kongens, Lyngby, Denmark
7. Center for Marine Biotechnology and Biomedicine, Scripps Institution of Oceanography, University of California San Diego, La Jolla, CA 92093, United States of America
8. Department of Computer Science and Engineering, University of California San Diego, La Jolla, CA 92093, USA.
9. Center for Microbiome Innovation, University of California San Diego, La Jolla, CA 92093, USA
10. Collaborative to Halt Antibiotic-Resistant Microbes (CHARM), Department of Pediatrics, UC San Diego, La Jolla, CA 92093

Correspondence

†To whom correspondence should be addressed:
Bernhard O. Palsson
University of California, San Diego
9500 Gilman Drive
La Jolla, CA 92093
palsson@ucsd.edu

Abstract

Evolving antibiotic resistant behavior of Healthcare-associated methicillin-resistant *Staphylococcus aureus* (HA-MRSA) USA100 strains are of major concern. They are resistant to a broad class of antibiotics like macrolides, aminoglycosides, fluoroquinolones, and many more. The selection of appropriate antibiotic susceptibility examination media is very important. Thus, we use bacteriological (CA-MHB) as well as physiological (R10LB) media to determine the effect of vancomycin on USA100 strains. The study includes the profiling behavior of HA-MRSA USA100 D592 and D712 strains in the presence of vancomycin through various high-throughput assays. The US100 D592 and D712 strains were characterized at sub-inhibitory concentrations through growth curves, RNA sequencing, bacterial cytological profiling, and exo-metabolomics high throughput experiments. The study reveals the vancomycin resistance behavior of USA100 strains in dual media conditions using wide-ranging experiments.

Background

The prevalence of the MRSA infections like bacteremia differs around the world and amongst the important cause of nosocomial infections worldwide [1]. Healthcare-associated MRSA (HA-MRSA) is a subset of MRSA strains that often circulate in healthcare settings such as hospitals, dialysis centers, etc [2–4]. The USA100 strain is the most common HA-MRSA with consistently high resistance to a wide range of antibiotics like macrolides, fluoroquinolones, and lincosamides [5,6]. Moreover, they are considered to display vancomycin-resistant and intermediate phenotypes [7]. Vancomycin was first approved to treat MRSA in the late 1980s. However, by the 1990's vancomycin-intermediate strains (VISA) had already begun to emerge. In 2002, the U.S. reported the first case of vancomycin-resistant *S. aureus* (VRSA) [8]. To understand the genetic and phenotypic basis for the emergence of this resistance, we collected multi-omic data on HA-MRSA strains, D592 (daptomycin-susceptible) and its descendent D712 (daptomycin-nonsusceptible) previously collected from a patient with prolonged and persistent MRSA bacteremia for 21 days [9,10]. Vancomycin is one of the few drugs that actually work daptomycin susceptible and daptomycin nonsusceptible strains. Thus, in the current study we are trying to understand the effects of vancomycin.

Cation-adjusted Mueller-Hinton broth (CAMHB) is a standard medium for quantitative procedures for susceptibility testing in microbiology labs worldwide [11]. CAMHB is commonly used for antibiotic susceptibility as it is enriched in divalent ions, Ca^{+2} and Mg^{+2}. The presence of divalent ions affects the stability of antibiotics or mode of action of antibiotics, which in turn greatly affects

the minimal inhibitory concentration (MIC) values. Roswell Park Memorial Institute (RPMI) 1640 media is amongst one of the best media to mimic human physiology [12–14].

The current study is focused on exploring the effect of vancomycin on HA-MRSA USA100 D712 and D592 strains in bacteriological (CA-MHB) and physiological (RPMI+10%LB) media. The MIC value of D592 decreased from 2□g/ml R10LB to 1□g/ml in CAMHB in the presence of vancomycin. Further, for the D712 strain, the MIC value decreased from 2□g/ml in R10LB to 0.96□g/ml in CAMHB. Though these MIC values fall below the clinically defined vancomycin resistance levels (add resistance levels here), vancomycin treatment was not unable to clear the bacteremia caused by these isolates [9]. Here we interrogated the response of HA-MRSA strain to the sub-inhibitory concentration of vancomycin using growth curves, RNA sequencing, bacterial cytological profiling (BCP), and exo-metabolomics (HPLC and LC-MS). Together, our data provide an in-depth look into vancomycin response by simultaneously tracking gene expression (RNA-seq), cell morphology (BCP), and changes in the chemical composition of the media (HPLC and LC/MS).

Methods

The methods used in the current study are validated in our previous papers [13,15].

Culture and Growth Conditions

We used standard bacteriological and physiological media to identify the effect of Vancomycin on USA100 strains. The standard bacteriological media includes Mueller-Hinton broth (Sigma-

Aldrich), and supplemented with 25 mg/L Ca^{2+} and 12.5 mg/L Mg2+ (CA-MHB). The physiological media including Roswell Park Memorial Institute 1640 (RPMI) as eukaryotic cell culture media (Thermo Fisher Scientific), which was supplemented by 10% LB (R10LB). The broth microdilution assay was performed to check the MIC of vancomycin in both the media condition. Both the USA100 HA-MRSA i.e. D592 and D712 were grown overnight. Further, overnight grown samples were diluted starting from OD600 of 0.01 through fresh media to OD600 of 0.4 at 37°C. The preculture was again diluted to OD600 of 0.01 by fresh media in the absence of vancomycin. Overall growth was monitored and OD600 readings were taken for 5.25 hr at every 45 min. Finally, the readings were collected in three biological replicates. The growth curve is provided as **Figure 1**. Concentrations of vancomycin shown in Figure 1 were chosen from a larger range of antibiotic concentrations based on the pattern of growth inhibition. In order to get reasonable and reproducible profiles in all downstream analyses, final assay concentrations were based on the constraint of no more than 50% growth inhibition relative to the untreated control of the strain of interest in the media of interest rather than multiples of the respective MIC. Preliminary growth curves were performed in at least biological duplicate, depending on the difficulty in finding reasonable growth inhibition, and final assay experiments were performed in biological duplicate. We were also interested in looking at overlapping concentrations for each strain, which is why there is an additional antibiotic concentration in the CA-MHB conditions but not in the RPMI+10%LB conditions.

cDNA library preparation and RNA sequencing

For RNA sequencing, the tubes containing 3mL samples were taken after 3 hours and added to a tube containing 6mL RNAprotect and centrifuged after incubation. The 3 hour time point for RNAseq was chosen to allow for approximately four doublings of the bacteria in the presence of vancomycin, providing time for the antibiotic to have a robust effect on the transcriptional and phenotypic response of the bacteria. The 'Quick RNA Fungal/Bacterial Microprep' kit (Zymo Research) was used for the RNA extraction from the pellet cells. During the RNA purification, the mechanical lysis was performed through Roche MagNa Lyser instrument, while DNA was removed through DNase I treatment. Illumina Ribo-Zero kit was used to remove the ribosomal RNA. The quality of RNA was checked through the Agilent Bioanalyzer instrument. Further, the cDNA library was constructed before sequencing through a KAPA Stranded RNA-seq Library Preparation Kit. Lastly, the RNA fragmentation, sequencing adapter ligation, and library amplification were done. The generated cDNA libraries were sent for Illumina sequencing on a HiSeq 4000 platform.

RNA sequencing analysis

For Illumina sequencing, the Phred quality scores were generated using Fastqc package [16]. The alignment of the raw reads were done for D712 and D592 genomes using Bowtie2 [17],[18] and FastQC [19] to calculate alignment percentage. Further the DESeq2 package was used to normalize the aligned reads to transcripts per million (TPM). Lastly, the technical validation was done through Principal component analysis (PCA) using sklearn package [20,21],[22]. The summary steps are provided in **Figure 2**.

DNA sequencing and genome assembly

The reference genome of D592 and D712 were sequenced using an Illumina Hiseq 4000 (paired-end, 100/100 bp reads) and Nanopore MinION to 50x and 60X coverage. Firstly, for the Illumina sequencing, the genomic DNA was prepared through Zymo Research Quick-DNA Fungal/Bacterial Microprep Kit. However, Kapa Biosystems HyprePlus kit was used to construct the libraries. Secondly, for the MinION sequencing, the genomic DNA was prepared through the CTAB method. Further, the Oxford Nanopore Rapid Barcoding Kit was used to construct the libraries. The quality control steps involved the removal of unincorporated primers, PCR primers, and adapters. The assembly step involves Unicycler 0.4.2 in the “default” mode for assembling 02 contigs (genome and plasmid). Lastly, the annotation was performed through the NCBI Prokaryotic Genome Annotation Pipeline (PGAP) v4.11.

Bacterial Cytological Profiling

After 3 hours of treatment, samples were removed for fluorescence microscopy, as previously described with slight modifications [23–25]. The 3 hour time point for BCP was chosen to allow for approximately four doublings of the bacteria in the presence of vancomycin, providing time for the antibiotic to have a robust effect on the transcriptional and phenotypic response of the bacteria. In brief, 8 uL treated samples were added to tubes containing 2 uL dye mix (10 ug/mL DAPI, 2.5 uM SYTOX Green, 60 uL/mL FM4-64 in 1x T-base). The samples were then spotted onto an agarose pad slide (20% media, 1.2% agarose) for microscopy. Imaging was performed on an Applied Precision DV Elite epifluorescence microscope with a CMOS camera, with excitation and exposure times were kept constant for all images (TRITC/Cy-5 = 0.025s, FITC/FITC = 0.01s, DAPI/DAPI = 0.015s).

FIJI (ImageJ 1.51w) and Adobe Photoshop (2015.1) were used to adjust deconvolved images to decrease background in FM4-64 and DAPI channels to ensure proper identification of cell and DNA objects. Raw and deconvolved images were then further processed using a custom CellProfiler 3.0 pipeline that individually thresholded and filtered DAPI and FM4-64 channels to obtain segmentation masks for key cellular features such as the cell membrane, DNA and entire cell, for a total of 5285 features [26,27]. Feature selection was applied prior to analysis to create a subset of relevant features and minimize redundancy. The summary of the processing steps is presented in **Figure 2**.

Untargeted Liquid Chromatography Mass Spectrometry Data Acquisition

At the same time that samples of HA-MRSA USA100 D592 and D7128 were taken for OD600 measurements, approximately 400 µL was collected from each replicate of all growth conditions and syringe-filtered using a 0.22 µm disc filters (Millex-GV, MilliporeSigma) to remove the cells from the spent media. Exometabolomics data were taken every 45 minutes from T0 to T4.5hr, in coordination with the bacteria's doubling time. Filtered samples were immediately placed on dry ice and then stored at −80 °C until liquid chromatography-mass spectrometry (LC/MS) was performed. The LC/MS platform utilized an UltiMate3000 HPLC system (Thermo Scientific) paired to a Maxis Impact (Bruker Daltonics) quadrupole-time-of-flight mass spectrometer. Filtered media from the RPMI+10% LB cultures were injected onto the LC at a volume of 5µl, and filter media from CA-MHB cultures were injected at a volume of 2 µl. All samples were injected onto a Kinetex 2.6µm polar-C18 reverse-phase column (Phenomenex). The column temperature was maintained at 30 °C.

For chromatographic separations, mobile phase A was LC/MS grade water modified with 0.1% formic acid and mobile phase B was LC/MS grade acetonitrile modified with 0.1% formic acid. Samples were injected at 95% A/5% B and at 1 minute the gradient was ramped to 65%A/35%B over the next 4 minutes. The solvent composition was stepped-up to 0%A/100%B and held for 1 minute before being restored to 95%A/5% for equilibration prior to injection of the subsequent sample.

Elence from the HPLC was sprayed into the Maxis Impact mass spectrometer via an Apollo II electrospray ionization source. The mass spectrometer was controlled using otofControl v4.0.15 and the LC/MS sequence program was controlled using Hystar v3.2. During sample introduction into the mass spectrometer, the ESI source was configured to have a nebulizer gas pressure of 2 bar, drying gas flow rate of 9 liters/minute, and a drying gas temperature of 200°C. The mass spectrometer's inlet capillary voltage was set at 3500 volts with an endplate offset of 500 volts. The mass spectrometer ion transfer optics were set to the following: Ion funnel 1 250 Vpp (volts peak-to-peak), ion funnel 2 250Vpp, transfer hexapole RF 100 Vpp, quadrupole ion energy 5 eV (electron volts), and collision quadrupole energy of 5 eV. The collision quadrupole RF was stepped across four voltages per scan: 450, 550, 800, 1100 Vpp. The collision cell transfer time was stepped across four values per scan: 70, 75, 90 and 95 μsecs. TOF pre-pulse storage was fixed at 7.0 μsecs. The mass spectrometer scan rate was fixed at 3Hz.

Prior to analysis, the mass spectrometer was externally calibrated using a sodium formate solution which was prepared by adding 100 μl of 1 M NaOH and 0.2% formic acid into 9.9mL of a 50%/50% water and isopropanol mixture. During mass spectrometric data acquisition, hexakis (1H,1H,2H-difluoroethoxy)-phosphazene (SynQuest Labs, Inc.) was used as a "lock mass" internal calibrant (positive mode: m/z 622.028960; $C_{12}H_{19}F_{12}N_3O_6P_3+$; negative polarity: ion m/z

556.001951 $C_{10}H_{15}F_{10}N_3O_6P_3$–). Subsequent to data acquisition, the lock mass was used to apply a linear mass correction to all mass spectra using Bruker Daltonics Compass Data Analysis software (ver. 4.3.110). Lock mass corrected data files were converted from the proprietary format (.d) to the mzXML open data format. All data herein were deposited to MassIVE (http://massive.ucsd.edu). The brief methodology is provided in Figure 2.

Targeted High-Performance Liquid Chromatography

The high-performance liquid chromatography (HPLC) experiment was performed for detection of organic acids and carbohydrates. All the samples were filtered and collected for every 45 minutes as discussed above. The 1260 Infinity series (Agilent Technologies) high-performance liquid chromatography (HPLC) system was used to load the samples using Aminex HPX-87H column (Bio-Rad Laboratories) and a refractive index detector. Overall system was run through ChemStation software. The HPLC grade water buffered with 5 mM sulfuric acid (H2SO4) was used as single mobile phase. At the temperature of 45 °C, the 10 □L samples were injected with the flow rate of 0.5 mL/minute. The compounds like ethanol, acetate, lactate, glucose, succinate, and pyruvate were determined by comparing the retention time with their standard graph. Finally, the resulting chromatograms and peak area integration were generated using ChemStation. The chromatograms and peaks were compared with the standard graph to detect the concentration of each compound within the samples. These final concentration values were deposited to the MassIVE database. The procedure of HPLC is depicted in **Figure 2,** while the measured concentration of two carbon sources is provided in **Figure 3**.

Results and Validation

All the multidimensional data of RNA sequencing, bacterial cytological profiling, and exo-metabolomics has been passed through quality control processes. Among all the QC steps, first is the batch effect analysis as shown in "exclusion criteria" section. Further, all the reads of RNAseq, BCP, and exometabolomics were scanned for the reproducibility as shown in the individual sections.

Exclusion Criteria

The data of 1.4ug/mL sub-inhibitory concentration for CA-MHB on the D712 strain has been excluded from all studies because the reproducibility between the samples was too low. The data and its result can be accessed in a public repository or available upon request.

DNA sequencing

The reference genome *S. aureus* D712 (VFJD01000001.1) [28] and D592 (NZ_CP035791.1) [29] were submitted to NCBI. The genome coverage of reference genome *S. aureus* D712 is 60X with the final genome size is 2,825,989 bp, While for the *S. aureus* D592, the genome coverage and genome size are 50X and 2,820,177bp respectively. The D712 is an evolved strain of D592. Both D592 and D712 strains were collected from the same patient upon pre-daptomycin treatment and after daptomycin treatment, respectively.

RNA sequencing

Firstly, the quality control steps were performed to remove unincorporated primers, adaptors, and detectable PCR primers. Further, the sequencing reads shows the average Phred score in D592 and

D712 is >38.1 and >39.1 and respectively. The raw fastq files were uploaded at the NCBI BioProject web platform. The alignment of reads with the reference genome in D592 and D712 gives an alignment score of 98.55% and 99.52% correspondingly. The RNAseq results are shown in **Figure 4**.

Bacterial Cytological profiling

The manual screening was performed on the image segmentation process of the CellProfiler. We scanned the representative images through manual curation of the accurate cell, object traces, and measurements. Further, the cell outlines were overlaid on the corresponding related structures e.g. DNA, cell membrane, cell wall, etc. Finally, the resultant files for all the cellular features were uploaded to the MassIVE repository. A representation of the image analysis pipeline for the BCP data is shown in **Figure 5**.

Untargeted Liquid Chromatography Mass Spectrometry data acquisition

For each sample, the reproducibility of global retention time and ion intensity were calculated by comparing the base peak chromatogram (BPCs) and multiple extracted ion chromatograms (EICs). The BPCs of each experimental replicate were obtained by comparing the peak intensity and reproducibility of retention time. While the EICs of the molecules were evaluated using the peak area and retention time drift of <0.1 minutes and <15% correspondingly.

Data Records

The growth-rate data is available on Figshare [30] , while BCP, HPLC, and Mass spectrometry data have been deposited on MassIVE repository [31]. Complete RNAseq pipeline can be found

at Figshare [32], Fastq files of each run have been deposited on NCBI- BioProject [33]. However, the overall summarized statistics of RNAseq is available on Figshare [34].

Reuse Potential

Systems analysis and machine learning methods are increasingly being utilized to understand antibiotic resistance. The models generated by these methods however, require a large volume of high quality and well curated data to be parameterized properly. The data and the associated metadata presented herein will be valuable is parameterizing many different types of models that can be used to query the underlying causes of antibiotic resistance.

The increasingly ubiquitous RNA-sequencing data has been utilized to predict the transcriptional regulatory networks in *S. aureus* where antibodies for most regulators are not readily available [35]. It has additionally been used to predict fitness and sensitivities to different antibiotics in different pathogens [36]. In parallel to RNA-sequencing based approaches, metabolic modeling has also come at the forefront of understanding resistance mechanisms. Combining metabolic models with machine learning methods have revealed metabolic pathways crucial for antibiotic resistance [37,38]. These metabolic models can be further parameterized to condition specific states with the presented exo-metabolomics data [39–41].

Lastly, the BCP data has been independently utilized to predict the various cellular subsystems that are affected by any given (known or unknown) antibiotic[24]. Together, these datasets will be

utilized to generate models that inform us about transcriptional regulation, metabolic shifts and morphological changes in response to antibiotic resistance.

Code availability

The complete RNAseq pipeline used in the analysis of RNAseq data is available on Figshare [32].

Acknowledgments

We thank Anand Sastry for helping build the RNA sequencing analysis pipeline. This research was supported by NIH NIAID grant (1-U01-AI124316).

Author Contributions

A.R. compiled, analyzed results and wrote Data Descriptor, Methods and Technical Validation S.P. analyzed RNA sequencing data and wrote Methods H.T. performed growth experiments, analyzed BCP data, and wrote Methods M.M. analyzed HPLC data and wrote Data Descriptor, Methods R.S. prepared samples for RNA sequencing and wrote Methods C.A.O. prepared samples for HPLC and wrote Methods A.L. performed growth experiments, analyzed BCP data, and wrote Methods Y.S. analysed HPLC data. N.D. performed preliminary growth and MIC experiments. A.V. performed growth experiments. J.S. wrote Methods. S.M.D. performed growth experiments.

Competing Interests

The authors declare no competing interests.

References

1. Hassoun A, Linden PK, Friedman B. Incidence, prevalence, and management of MRSA bacteremia across patient populations-a review of recent developments in MRSA management and treatment. *Crit Care*. 21:2112017;

2. Thompson RL, Cabezudo I, Wenzel RP. Epidemiology of nosocomial infections caused by methicillin-resistant Staphylococcus aureus. *Ann Intern Med*. 97:309–171982;

3. Chambers HF, Deleo FR. Waves of resistance: Staphylococcus aureus in the antibiotic era. *Nat Rev Microbiol*. 7:629–412009;

4. Kong EF, Johnson JK, Jabra-Rizk MA. Community-Associated Methicillin-Resistant Staphylococcus aureus: An Enemy amidst Us. *PLoS Pathog*. 12:e10058372016;

5. Richter SS, Heilmann KP, Dohrn CL, Riahi F, Costello AJ, Kroeger JS, et al.. Activity of ceftaroline and epidemiologic trends in Staphylococcus aureus isolates collected from 43 medical centers in the United States in 2009. *Antimicrob Agents Chemother*. 55:4154–602011;

6. David MZ, Cadilla A, Boyle-Vavra S, Daum RS. Replacement of HA-MRSA by CA-MRSA infections at an academic medical center in the midwestern United States, 2004-5 to 2008. *PLoS One*. 9:e927602014;

7. Roberts JC. Community-associated methicillin-resistant staphylococcus aureus epidemic clone USA100; more than a nosocomial pathogen. *Springerplus*. 2:1332013;

8. McGuinness WA, Malachowa N, DeLeo FR. Vancomycin Resistance in Staphylococcus aureus. *Yale J Biol Med*. 90:269–812017;

9. Sakoulas G, Okumura CY, Thienphrapa W, Olson J, Nonejuie P, Dam Q, et al.. Nafcillin enhances innate immune-mediated killing of methicillin-resistant Staphylococcus aureus. *J Mol Med* . 92:139–492014;

10. Dhand A, Bayer AS, Pogliano J, Yang S-J, Bolaris M, Nizet V, et al.. Use of antistaphylococcal beta-lactams to increase daptomycin activity in eradicating persistent bacteremia due to methicillin-resistant Staphylococcus aureus: role of enhanced daptomycin binding. *Clin Infect Dis*. 53:158–632011;

11. Koeth LM, King A, Knight H, May J, Miller LA, Phillips I, et al.. Comparison of cation-adjusted Mueller-Hinton broth with Iso-Sensitest broth for the NCCLS broth microdilution method. *J Antimicrob Chemother*. 46:369–762000;

12. Meerwein M, Tarnutzer A, Böni M, Van Bambeke F, Hombach M, Zinkernagel AS. Increased Azithromycin Susceptibility of Multidrug-Resistant Gram-Negative Bacteria on RPMI-1640 Agar Assessed by Disk Diffusion Testing. *Antibiotics*. Multidisciplinary Digital Publishing Institute; 9:2182020;

13. Poudel S, Tsunemoto H, Meehan M, Szubin R, Olson CA, Lamsa A, et al.. Characterization of CA-MRSA TCH1516 exposed to nafcillin in bacteriological and physiological media. *Sci Data*. 6:432019;

14. Cantor JR, Abu-Remaileh M, Kanarek N, Freinkman E, Gao X, Louissaint A Jr, et al.. Physiologic Medium Rewires Cellular Metabolism and Reveals Uric Acid as an Endogenous Inhibitor of UMP Synthase. *Cell*. 169:258–72.e172017;

15. Rajput A, Poudel S, Tsunemoto H, Meehan M, Szubin R, Olson CA, et al.. Profiling the effect of nafcillin on HA-MRSA D712 using bacteriological and physiological media. *Sci Data*. 6:3222019;

16. Turnidge JD. The pharmacodynamics of beta-lactams. *Clin Infect Dis*. 27:10–221998;

17. Monk JM: Staphylococcus aureus strain D712, whole genome shotgun sequencing project. GenBank. https://identifiers.org/ncbi/insdc:VFJD00000000.1 (2019).

18. Monk JM: Staphylococcus aureus strain 592 chromosome, complete genome. GenBank. GenBank; https://identifiers.org/ncbi/insdc:CP035791.1

19. Andrews S: A quality control tool for high throughput sequence data. FastQC. Accessed; http://www.bioinformatics.babraham.ac.uk/projects/fastqc/ (2010).

20. Langmead B, Salzberg SL. Fast gapped-read alignment with Bowtie 2. *Nat Methods*. 9:357–92012;

21. Leek JT, Johnson WE, Parker HS, Jaffe AE, Storey JD. The sva package for removing batch effects and other unwanted variation in high-throughput experiments. *Bioinformatics*. 28:882–32012;

22. Johnson WE, Li C, Rabinovic A. Adjusting batch effects in microarray expression data using empirical Bayes methods. *Biostatistics*. 8:118–272007;

23. Pedregosa F, Varoquaux G, Gramfort A, Michel V, Thirion B, Grisel O, et al.. Scikit-learn: Machine Learning in Python. *J Mach Learn Res*. 12:2825–302011;

24. Nonejuie P, Burkart M, Pogliano K, Pogliano J. Bacterial cytological profiling rapidly identifies the cellular pathways targeted by antibacterial molecules. *Proc Natl Acad Sci U S A*. 110:16169–742013;

25. Quach DT, Sakoulas G, Nizet V, Pogliano J, Pogliano K. Bacterial Cytological Profiling (BCP) as a Rapid and Accurate Antimicrobial Susceptibility Testing Method for Staphylococcus aureus. *EBioMedicine*. 4:95–1032016;

26. Carpenter AE, Jones TR, Lamprecht MR, Clarke C, Kang IH, Friman O, et al.. CellProfiler: image analysis software for identifying and quantifying cell phenotypes. *Genome Biol*. 7:R1002006;

27. Rodenacker K, Bengtsson E. A feature set for cytometry on digitized microscopic images. *Anal Cell Pathol*. 25:1–362003;

28. : ASM755669v1. NCBI-Assembly. NCBI-Assembly;

https://www.ncbi.nlm.nih.gov/assembly/GCA_007556695.1

29. : ASM420859v1. NCBI-Assembly. NCBI-Assembly; https://www.ncbi.nlm.nih.gov/assembly/GCA_004208595.1

30. Rajput A. Growth curve values of D712 and D592 strains. figshare;

31. Palsson BO. Staphylococcus aureus D592 & D712 +Vancomycin (LC/MS, HPLC, BPC data). MassIVE;

32. Rajput A. RNAseq workflow. figshare;

33. Palsson B: Response of Staphylococcus aureus USA100 strains to subinhibitory concentration of Vancomycin. BioProject. BioProject; https://www.ncbi.nlm.nih.gov/bioproject/638628

34. Rajput A. RNAseq summary. figshare;

35. Poudel S, Tsunemoto H, Seif Y, Sastry AV, Szubin R, Xu S, et al.. Revealing 29 sets of independently modulated genes in Staphylococcus aureus, their regulators, and role in key physiological response. *Proc Natl Acad Sci U S A*. 117:17228–392020;

36. Zhu Z, Surujon D, Ortiz-Marquez JC, Huo W, Isberg RR, Bento J, et al.. Entropy of a bacterial stress response is a generalizable predictor for fitness and antibiotic sensitivity. *Nat Commun*. 11:43652020;

37. Kavvas ES, Yang L, Monk JM, Heckmann D, Palsson BO. A biochemically-interpretable machine learning classifier for microbial GWAS. *Nat Commun*. 11:25802020;

38. Yang JH, Wright SN, Hamblin M, McCloskey D, Alcantar MA, Schrübbers L, et al.. A White-Box Machine Learning Approach for Revealing Antibiotic Mechanisms of Action. *Cell*. 177:1649–61.e92019;

39. Aurich MK, Paglia G, Rolfsson Ó, Hrafnsdóttir S, Magnúsdóttir M, Stefaniak MM, et al.. Prediction of intracellular metabolic states from extracellular metabolomic data. *Metabolomics*. 11:603–192015;

40. Ramon C, Gollub MG, Stelling J. Integrating--omics data into genome-scale metabolic network models: principles and challenges. *Essays Biochem*. Portland Press Ltd.; 62:563–742018;

41. Yurkovich JT, Palsson BO. Quantitative -omic data empowers bottom-up systems biology. *Curr Opin Biotechnol*. 51:130–62018;

Figure legends

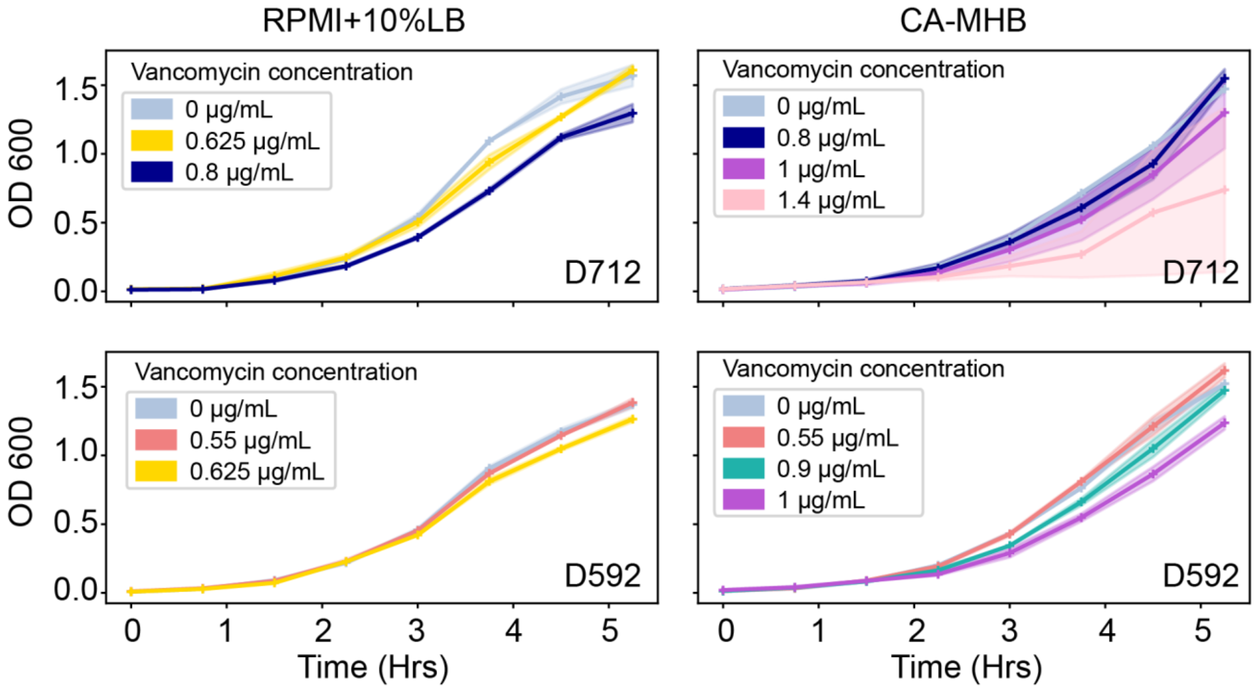

Figure 1. Growth curve for Staphylococcus aureus D592 and D712 strain in presence of vancomycin at various sub-inhibitory concentrations in CA-MHB and R10LB media.

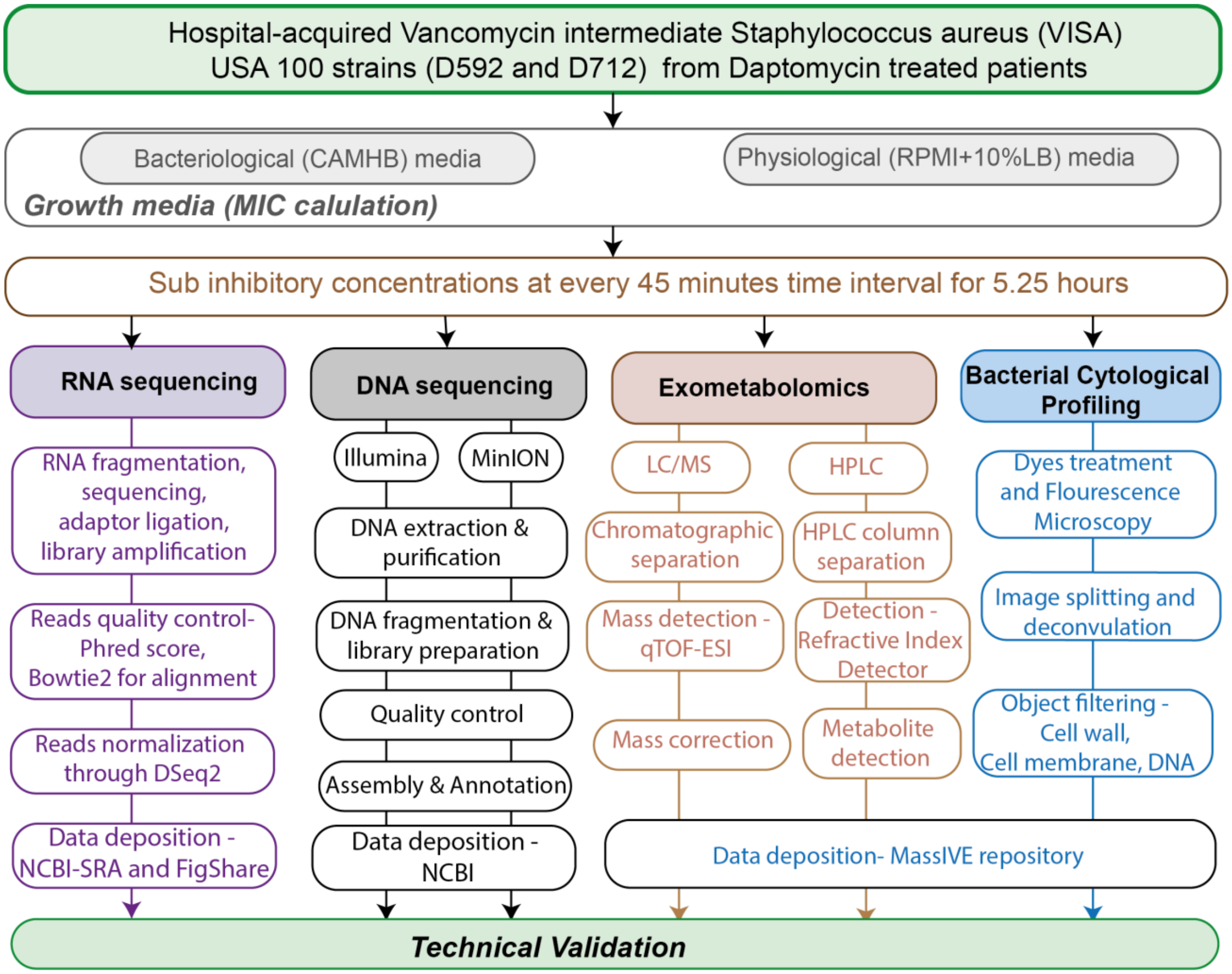

Figure 2. Diagram depicting the methodology of high throughput approaches used to profile the Staphylococcus aureus D592 and D712 in presence of vancomycin.

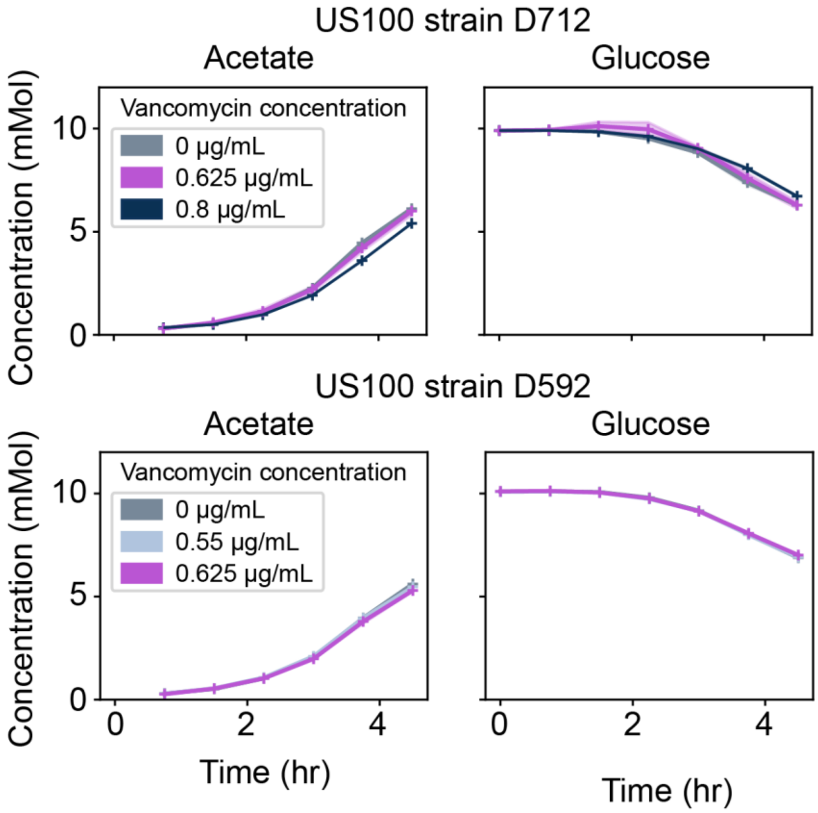

Figure 3. HPLC-derived quantitative time-course exo-metabolomics measurements for Staphylococcus aureus D592 and D712 cells exposed to various antibiotic concentrations in RPMI + 10%LB and CA-MHB. Here, we show the absolute calibrated concentrations of acetate and D-glucose in both media types.

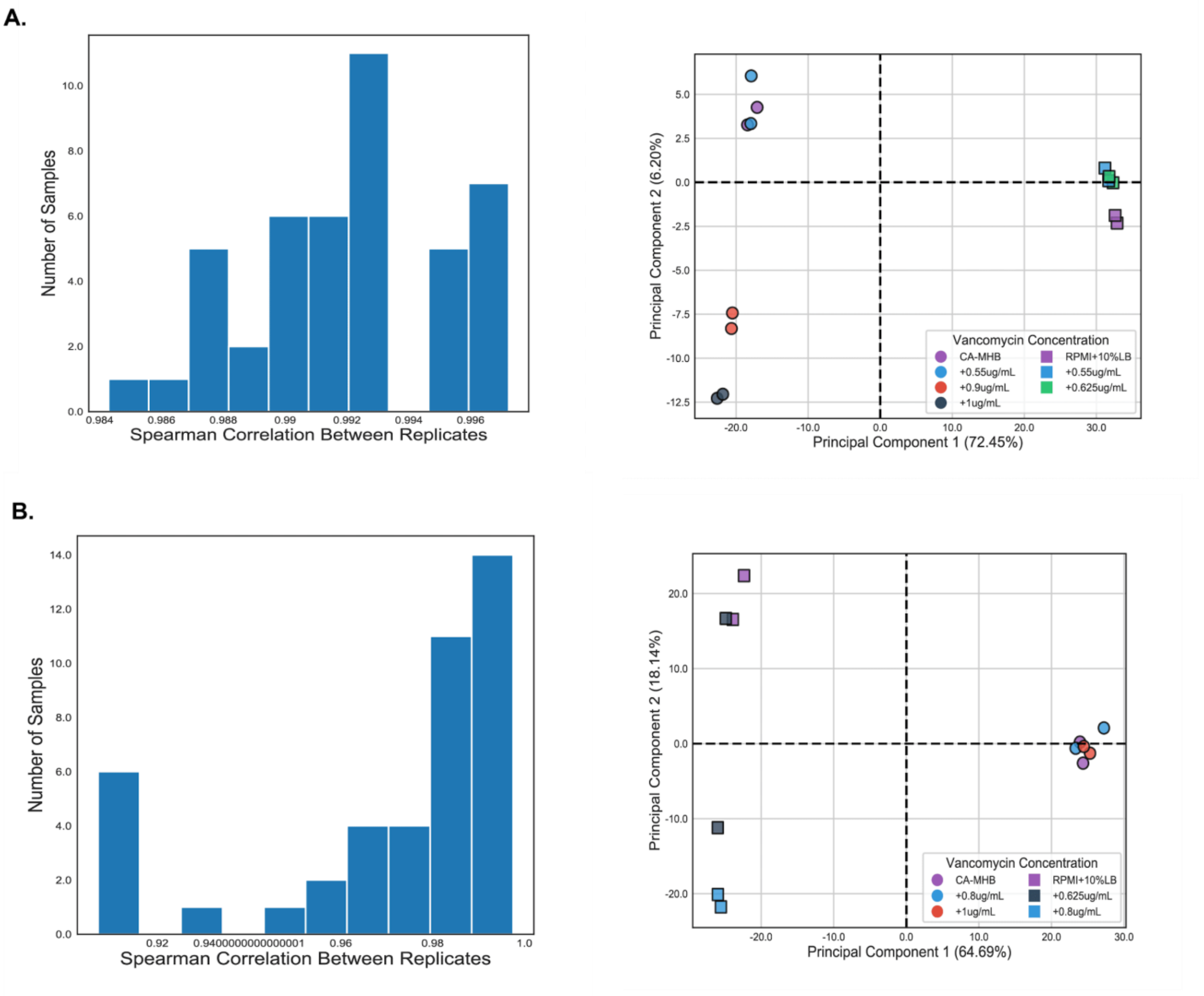

Figure 4. RNAseq results. A) Clustering of reads TPM as per Spearman's correlation coefficient and PCA plot for D592 strains B) Clustering of reads TPM as per Spearman's correlation coefficient and PCA plot for D712 strains.

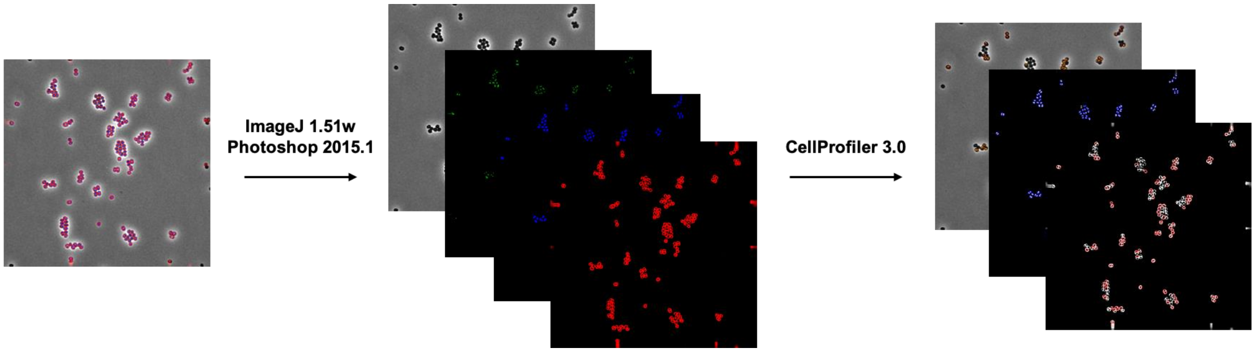

Figure 5. Depiction of image analysis pipeline for Bacterial cytological profiling of Staphylococcus aureus D592 and D712 in presence of vancomycin.

Cover letter
Click here to access/download;Manuscript;rajput-giga_cover_R1.pdf

UNIVERSITY OF CALIFORNIA, SAN DIEGO UCSD

BERKELEY • DAVIS • IRVINE • LOS ANGELES • MERCED • RIVERSIDE • SAN DIEGO • SAN FRANCISCO SANTA BARBARA • SANTA CRUZ

BERNHARD Ø. PALSSON
PROFESSOR
DEPARTMENT OF BIOENGINEERING - 0412
PFBH, ROOM 417
UNIVERSITY OF CALIFORNIA, SAN DIEGO

9500 GILMAN DRIVE
LA JOLLA, CALIFORNIA 92093-0412
TELEPHONE: (858) 534-5668
FAX: (858) 822-3120
palsson@ucsd.edu
http://systemsbiology.ucsd.edu

October 20, 2020

Dear Dr. Zauner,

We would like to submit the revised manuscript "*Identifying the effect of vancomycin on HA-MRSA strains using bacteriological and physiological media*" in the "Data Note" section of the "Giga Science" journal (GIGA-D-20-00193). The study contains the multidimensional data generated from various approaches to characterize the effect of Vancomycin on hospital-acquired (HA) USA100 'D592' and 'D712' *Staphylococcus aureus* strain in both bacteriological and physiological media.

As per the editor's concern, we have provided a detailed methodology of data production, result validation, and reuse potential in the manuscript. Present data would be of great importance for various studies like bacterial cytological profiling, RNAseq studies, etc. We have also addressed all the issues raised by the reviewer regarding the work. The overall objective of the study is to explore the effect of vancomycin on the D592 (daptomycin-susceptible) and D712 (daptomycin-nonsusceptible) strains. As vancomycin is one of the few drugs that actually work daptomycin susceptible and daptomycin nonsusceptible strains. Thus, we checked the effect of both strains on the bacteriological (CA-MHB) and physiological media (RPMI+10%LB) through high throughput experiments like DNA sequencing, RNA sequencing, growth curves, bacterial cytological profiling (BCP), and exo-metabolomics (HPLC and LC-MS).

All the high-quality raw data is publicly available. The growth curve data is in FigShare; DNAseq and RNAseq data were submitted in NCBI platforms, and FigShare; BCP and exo-metabolomics (HPLC and LC-MS) experiments output is submitted in the MaSSIVE database.

We hope that you would consider our work worth publishing in your journal. Please do not hesitate to contact me with any further questions.

Sincerely,

Bernhard Palsson

Click here to access/download
Supplementary Material
Reviewers comments.pdf

Manuscript changes

Click here to access/download
Supplementary Material
VanD712_D592_R1_Changes.docx
