# Supplementary material for: Identifying the effect of vancomycin on health care–associated methicillin-resistant Staphylococcus aureus strains using bacteriological and physiological media
Source: Gigascience. 2021 Jan 9;10(1):giaa156. doi: 10.1093/gigascience/giaa156 (PMC7794652; doi:10.1093/gigascience/giaa156)
Supplement: giaa156_Response_to_Reviewer_Comments_Original_Submission [file giaa156_response_to_reviewer_comments_original_submission.pdf]

Author's Response To Reviewer Comments

Close

Reviewer #1:
The authors have addressed my concerns. I have no further questions.
Response:
We are very thankful to the reviewer for accepting our manuscript for the publication.

Reviewer #2:
Author response: Concentrations of vancomycin shown in Figure 1 were chosen from a larger range of antibiotic concentrations based on the pattern of growth inhibition. In order to get reasonable and reproducible profiles in all downstream analyses, final assay concentrations were based on the constraint of no more than 50% growth inhibition relative to the untreated control of the strain of interest in the media of interest rather than multiples of the respective MIC. Preliminary growth curves were performed in at least biological duplicate, depending on the difficulty in finding reasonable growth inhibition, and final assay experiments were performed in biological duplicate. We were also interested in looking at overlapping concentrations for each strain, which is why there is an additional antibiotic concentration in the CA-MHB conditions but not in the RPMI+10%LB conditions.

My Response. I now understand the rational for selecting different concentrations for each strain, however it is not clear that these concentrations are then used for each of the experimental processes. I suggest a few sentences to clarify this point.
'For all subsequent experiments, and based on preliminary growth analysis, D592 was exposed to 0, 0.55, 0.9, and 1.0 ug/ml of vancomycin in CA MHB media, and 0, 0.55, and 0.625 ug/ml in RPMI. D712 was exposed 0, 0.8, 1, 1.4 ug/ml in CA MHB and 0, 0.625, 0.8 ug/ml" in RPMI. Or something to this effect
Similarly, it is not obvious how many biological replicates was performed for each experiment (which I specifically requested in the previous round). Sure, if you look closely at figure 4, you see that the RNA-seq was performed for two biological replicates, but this should be clearly stated in the text. It is not clear how many biological replicates for the BCP or the metabolomics are available for analysis.

Response:
We have incorporated the different concentrations of vancomycin in the “method” section of the manuscript. We used two biological replicates in all the experiments like BCP, metabolomics, and RNAseq. We have also updated this information in the respective sections of the manuscript.

Author Response: The 3 hour time point for RNAseq and BCP was chosen to allow for approximately four doublings of the bacteria in the presence of vancomycin, providing time for the antibiotic to have a robust effect on the transcriptional and phenotypic response of the bacteria.

My Response. OK

Author Response: To understand the genetic and phenotypic basis for the emergence of this resistance, we collected multi-omic data on HA-MRSA strains, D592 (daptomycin-susceptible) and its descendent D712 (daptomycin-nonsusceptible) previously collected from a patient with prolonged and persistent MRSA bacteremia for 21 days (PMID: 21690622). As, vancomycin is one of the last few drugs that actually work daptomycin susceptible and daptomycin nonsusceptible strains. Thus, in the current study we are trying to understand the effects of vancomycin.
The MIC value of D592 decreased from 2g/ml R10LB to 1g/ml in CAMHB in the presence of vancomycin. Further, for the D712 strain, the MIC value decreased from 2g/ml in R10LB to 0.96g/ml in CAMHB. Though these MIC values fall below the clinically defined vancomycin resistance levels (add resistance levels here), vancomycin treatment was not unable to clear the bacteremia caused by these isolates.

My Response. The reference provided (PMID: 21690622) reports an MIC of 3 ug/ml for D712 to

vancomycin (I presume). This is different to the MIC reported in the current submission. It is strange that the MIC in CAMHB for each strain is ~1ug/ml, yet each strain grows very happily at that concentration (figure 1). Is there an explanation for this? Perhaps the MICs should be retested to determine if they fit with the previous report (PMID: 21690622) and the data from the growth curves.

Response:
The MICs have been retested and are within range of those listed in the submitted manuscript for both strains and media types. The difference in the observed MIC values and the concentrations used in the growth curves can be attributed to the difference in the growth conditions. MICs were determined using CLSI guidelines for broth microdilution, including cell inoculum of ~5 x 105 CFU/mL and an incubation time of 18-24hrs. The growth curves, in contrast, have a starting inoculum of ~8 x 106 CFU/mL and a much shorter incubation time. Additionally, while the MICs plates are shaken at ~225 rpm, the growth curves were done in tubes containing a stir bar, and thus have much higher levels of aeration, which contributes to faster growth of the bacteria. These differences can account for the decreased sensitivity of the strains in the growth curves compared to the MIC values. The concentrations selected for the published growth curves and subsequent sampling were determined using wider ranges of concentrations around the MIC in biological duplicate, with the constraint of no more than 50% growth inhibition relative to the untreated control.

Author Response (related to proofreading): The following concerns have been addressed.

My Response: The text would benefit from additional proofreading - example, "(add resistance levels here)" which appeared in both the text, and the response to review.

Response:
The proofreading has been done.

Author Response: Even though the proportion of MRSA isolates in Europe has decreased over time, 7 of the 29 European Union countries still report 25% or more of invasive S. aureus isolates as MRSA (https://urldefense.com/v3/__https://www.ecdc.europa.eu/en/publications-data/antimicrobial-resistance-surveillance-europe-2014__;!!Mih3wA!XE6HQdniqYcggALpmGjun7SHRT0sQXcEbjiwFFLsJKmTvirDHv78t4Cz9dUltJE$). However, we have modified the wordings of the highlighted sentence.

My Response: the initial submission said that ' majority…worldwide'. 7 of 29 countries is not a majority, and 25% is also not a majority.

Response:
We agree with the reviewer. This point has been updated in the manuscript. The word “majority” has been removed.

Author Response: USA100 strains are typically associated with HA-MRSA. They already constituted a larger proportion of reported MRSA isolates in the earlier years of the study in eastern US states (PMID: 24755631; 27272665). Therefore, we used USA100 strains in the current study.

My Response: I still believe "The USA100 strain is the most common HA-MRSA…" should be changed or deleted. Similar to response above, this is not a study about MRSA epidemiology.
The text still states that vancomycin was first approved to treat MRSA in the late 1980s. This isn't true.

Response:
As per the reviewer’s concern, we have changed the portion of the manuscript.
“The USA100 strain is an HA-MRSA that shows high resistance to a wide range of antibiotics like macrolides, fluoroquinolones, and lincosamides. Since the last four decades, vancomycin has been the antibiotic of choice to treat MRSA. However, by the 1990s vancomycin-intermediate strains (VISA) had already begun to emerge (23511300).”

Author Response (pertaining to Figure 3): The 'pyruvate' and 'lactate' were included by mistake. The following concern has been updated.

My Reviewer. OK. However, I now realize that the legend says that the graphs show data using RPMI+10%LB and CA-MHB, but it can only be one of those. Both media should be included, so that it is

consistent with other figures.

Response:
We didn't add the CA-MHB data in Figure 3 because there wasn't much change in glucose or acetate concentration. However, we added the note regarding the same in the manuscript as well as the figure legend.
"The HPLC time-course exo-metabolomics measurements for S. aureus D592 and D712 cells in presence of different concentrations of the vancomycin in CA-MHB media wasn't shown because the differences were too slight."
For the reviewer's concern we are providing the figure below with both CAMHB and R10LB media.

Figure. HPLC-derived quantitative time-course exo-metabolomics measurements for Staphylococcus aureus D592 and D712 cells exposed to various antibiotic concentrations in RPMI + 10%LB and CA-MHB. Here, we show the absolute calibrated concentrations of acetate and D-glucose in both media types.

Close
