# Supplementary material for: Identifying the effect of vancomycin on health care–associated methicillin-resistant Staphylococcus aureus strains using bacteriological and physiological media
Source: Gigascience. 2021 Jan 9;10(1):giaa156. doi: 10.1093/gigascience/giaa156 (PMC7794652; doi:10.1093/gigascience/giaa156)
Supplement: giaa156_Response_to_Reviewer_Comments_Revision_1 [file giaa156_response_to_reviewer_comments_revision_1.pdf]

Author's Response To Reviewer Comments

Close

Response to Reviewers

1. Figshare citations:

A couple of data citations need more details (e.g. references # 31, 33, 35). Please refer to our citation style in our "information for authors". If available, always include the individual DOI (digital object identifier) of the (e.g. figshare) dataset; Only if the datasets don't have DOIs, include the URL.
Response:
We have included the DOIs in references#31, 33, 35.

2. GigaDB: We can also host supporting data in our own repository, GigaDB. Our data curators will contact you shortly to discuss this. If applicable, GigaDB datasets should be cited in the same way as the figshare repos, i.e. including a DOI.
Response:
We have included the DOIs of the figshare in the manuscript.

3. There's a mention of "... data available upon request" in the section "exclusion criteria". Please delete this - all data should be made available in public repositories prior to publication.
Response:
This concern has been updated.

4. Regarding the section "Data records":
4a title: Please rename this section, the title should be "Availability of supporting data"
4b Please make sure to include all relevant accession numbers in this section (NCBI for sequencing data, MassIVE for MAssSpec etc).
Response:
We have updated the "Data records" as "Availability of supporting data". As well as include the relevant accession numbers for the NCBI and MassIVE repositories.

5 You mention the availability of the pipeline code, which is great. Can you attach an OSI-approved licence to this code, and mention the licence in the repository and in the manuscript? (OSI licences: https://urldefense.com/v3/__https://opensource.org/licenses__;!!Mih3wA!VFfYuEwyva41mBVGSRshA-ai1HlXlGx4SnrNizKebKZjd_YLypTxd6GkJS7yfKQ$).
Response:
We have updated the license as CC BY 4.0 license in the manuscript.

6 Please include ORCIDs for all co-authors on the title page, if available.
Response:
We have included the ORCIDs of the authors on the title page.

We have updated the abstract section into three subsections i.e. "Background", "Findings", and "Conclusions".

Close
