# Supplementary material for: Identifying the effect of vancomycin on health care–associated methicillin-resistant Staphylococcus aureus strains using bacteriological and physiological media
Source: Gigascience. 2021 Jan 9;10(1):giaa156. doi: 10.1093/gigascience/giaa156 (PMC7794652; doi:10.1093/gigascience/giaa156)
Supplement: giaa156_Reviewer_2_Report_Original_Submission — David R. Cameron -- 11/16/2020 Reviewed [file giaa156_reviewer_2_report_original_submission.pdf]

Reviewer Report

Title: Identifying the effect of vancomycin on HA-MRSA strains using bacteriological and physiological media

Version: Original Submission **Date:** 11/16/2020

Reviewer name: David R. Cameron

Reviewer Comments to Author:

Author response: Concentrations of vancomycin shown in Figure 1 were chosen from a larger range of antibiotic concentrations based on the pattern of growth inhibition. In order to get reasonable and reproducible profiles in all downstream analyses, final assay concentrations were based on the constraint of no more than 50% growth inhibition relative to the untreated control of the strain of interest in the media of interest rather than multiples of the respective MIC. Preliminary growth curves were performed in at least biological duplicate, depending on the difficulty in finding reasonable growth inhibition, and final assay experiments were performed in biological duplicate. We were also interested in looking at overlapping concentrations for each strain, which is why there is an additional antibiotic concentration in the CA-MHB conditions but not in the RPMI+10%LB conditions.
My Response. I now understand the rational for selecting different concentrations for each strain, however it is not clear that these concentrations are then used for each of the experimental processes. I suggest a few sentences to clarify this point.
'For all subsequent experiments, and based on preliminary growth analysis, D592 was exposed to 0, 0.55, 0.9, and 1.0 ug/ml of vancomycin in CA MHB media, and 0, 0.55, and 0.625 ug/ml in RPMI. D712 was exposed 0, 0.8, 1, 1.4 ug/ml in CA MHB and 0, 0.625, 0.8 ug/ml" in RPMI. Or something to this effect
Similarly, it is not obvious how many biological replicates was performed for each experiment (which I specifically requested in the previous round). Sure, if you look closely at figure 4, you see that the RNA-seq was performed for two biological replicates, but this should be clearly stated in the text. It is not clear how many biological replicates for the BCP or the metabolomics are available for analysis.
Author Response: The 3 hour time point for RNAseq and BCP was chosen to allow for approximately four doublings of the bacteria in the presence of vancomycin, providing time for the antibiotic to have a robust effect on the transcriptional and phenotypic response of the bacteria.
My Response. OK
Author Response: To understand the genetic and phenotypic basis for the emergence of this resistance, we collected multi-omic data on HA-MRSA strains, D592 (daptomycin-susceptible) and its descendent D712 (daptomycin-nonsusceptible) previously collected from a patient with prolonged and persistent MRSA bacteremia for 21 days (PMID: 21690622). As, vancomycin is one of the last few drugs that actually work daptomycin susceptible and daptomycin nonsusceptible strains. Thus, in the current study we are trying to understand the effects of vancomycin.
The MIC value of D592 decreased from 2ð□›□g/ml R10LB to 1ð□›□g/ml in CAMHB in the presence of vancomycin. Further, for the D712 strain, the MIC value decreased from 2ð□›□g/ml in R10LB to

0.96ð›g/ml in CAMHB. Though these MIC values fall below the clinically defined vancomycin resistance levels (add resistance levels here), vancomycin treatment was not unable to clear the bacteremia caused by these isolates.

My Response. The reference provided (PMID: 21690622) reports an MIC of 3 ug/ml for D712 to vancomycin (I presume). This is different to the MIC reported in the current submission. It is strange that the MIC in CAMHB for each strain is ~1ug/ml, yet each strain grows very happily at that concentration (figure 1). Is there an explanation for this? Perhaps the MICs should be retested to determine if they fit with the previous report (PMID: 21690622) and the data from the growth curves.

Author Response (related to proofreading): The following concerns have been addressed.

My Response: The text would benefit from additional proofreading - example, "(add resistance levels here)" which appeared in both the text, and the response to review.

Author Response: Even though the proportion of MRSA isolates in Europe has decreased over time, 7 of the 29 European Union countries still report 25% or more of invasive S. aureus isolates as MRSA (https://www.ecdc.europa.eu/en/publications-data/antimicrobial-resistance-surveillance-europe-2014). However, we have modified the wordings of the highlighted sentence.

My Response: the initial submission said that 'MRSA makes up the majority…worldwide'. 7 of 29 countries is not a majority, and 25% is also not a majority.

Author Response: USA100 strains are typically associated with HA-MRSA. They already constituted a larger proportion of reported MRSA isolates in the earlier years of the study in eastern US states (PMID: 24755631; 27272665). Therefore, we used USA100 strains in the current study.

My Response: I still believe "The USA100 strain is the most common HA-MRSA…" should be changed or deleted. Similar to response above, this is not a study about MRSA epidemiology.

The text still states that vancomycin was first approved to treat MRSA in the late 1980s. This isn't true.

Author Response (pertaining to Figure 3): The 'pyruvate' and 'lactate' were included by mistake. The following concern has been updated.

My Reviewer. OK. However, I now realize that the legend says that the graphs show data using RPMI+10%LB and CA-MHB, but it can only be one of those. Both media should be included, so that it is consistent with other figures.

Level of Interest

Please indicate how interesting you found the manuscript: Choose an item.

Quality of Written English

Please indicate the quality of language in the manuscript: Choose an item.

Declaration of Competing Interests

Please complete a declaration of competing interests, considering the following questions:

- Have you in the past five years received reimbursements, fees, funding, or salary from an organisation that may in any way gain or lose financially from the publication of this manuscript, either now or in the future?
- Do you hold any stocks or shares in an organisation that may in any way gain or lose financially from the publication of this manuscript, either now or in the future?
- Do you hold or are you currently applying for any patents relating to the content of the manuscript?
- Have you received reimbursements, fees, funding, or salary from an organization that holds or has applied for patents relating to the content of the manuscript?
- Do you have any other financial competing interests?
- Do you have any non-financial competing interests in relation to this paper?

If you can answer no to all of the above, write 'I declare that I have no competing interests' below. If your reply is yes to any, please give details below.

I declare I have no competing interests.

I agree to the open peer review policy of the journal. I understand that my name will be included on my report to the authors and, if the manuscript is accepted for publication, my named report including any attachments I upload will be posted on the website along with the authors' responses. I agree for my report to be made available under an Open Access Creative Commons CC-BY license (http://creativecommons.org/licenses/by/4.0/). I understand that any comments which I do not wish to be included in my named report can be included as confidential comments to the editors, which will not be published.

Choose an item.

To further support our reviewers, we have joined with Publons, where you can gain additional credit to further highlight your hard work (see: https://publons.com/journal/530/gigascience). On publication of this paper, your review will be automatically added to Publons, you can then choose whether or not to claim your Publons credit. I understand this statement.

Yes Choose an item.
